# A role for domain I of the hepatitis C virus NS5A protein in virus assembly

**DOI:** 10.1371/journal.ppat.1006834

**Published:** 2018-01-19

**Authors:** Chunhong Yin, Niluka Goonawardane, Hazel Stewart, Mark Harris

**Affiliations:** School of Molecular and Cellular Biology, Faculty of Biological Sciences, and Astbury Centre for Structural Molecular Biology, University of Leeds, Leeds, United Kingdom; The University of Chicago, UNITED STATES

## Abstract

The NS5A protein of hepatitis C virus (HCV) plays roles in both virus genome replication and assembly. NS5A comprises three domains, of these domain I is believed to be involved exclusively in genome replication. In contrast, domains II and III are required for the production of infectious virus particles and are largely dispensable for genome replication. Domain I is highly conserved between HCV and related hepaciviruses, and is highly structured, exhibiting different dimeric conformations. To investigate the functions of domain I in more detail, we conducted a mutagenic study of 12 absolutely conserved and surface-exposed residues within the context of a JFH-1-derived sub-genomic replicon and infectious virus. Whilst most of these abrogated genome replication, three mutants (P35A, V67A and P145A) retained the ability to replicate but showed defects in virus assembly. P35A exhibited a modest reduction in infectivity, however V67A and P145A produced no infectious virus. Using a combination of density gradient fractionation, biochemical analysis and high resolution confocal microscopy we demonstrate that V67A and P145A disrupted the localisation of NS5A to lipid droplets. In addition, the localisation and size of lipid droplets in cells infected with these two mutants were perturbed compared to wildtype HCV. Biophysical analysis revealed that V67A and P145A abrogated the ability of purified domain I to dimerize and resulted in an increased affinity of binding to HCV 3’UTR RNA. Taken together, we propose that domain I of NS5A plays multiple roles in assembly, binding nascent genomic RNA and transporting it to lipid droplets where it is transferred to Core. Domain I also contributes to a change in lipid droplet morphology, increasing their size. This study reveals novel functions of NS5A domain I in assembly of infectious HCV and provides new perspectives on the virus lifecycle.

## Author summary

Hepatitis C virus infects 170 million people worldwide, causing long term liver disease. Recently new therapies comprising direct-acting antivirals (DAAs), small molecule inhibitors of virus proteins, have revolutionised treatment for infected patients. Despite this, we have a limited understanding of how the virus replicates in infected liver cells. Here we identify a previously uncharacterised function of the NS5A protein–a target for one class of DAAs. NS5A is comprised of three domains–we show that the first of these (domain I) plays a role in the production of new, infectious virus particles. Previously it was thought that domain I was only involved in replicating the virus genome. Mutations in domain I perturb dimer formation, enhanced binding to the 3’ end of the virus RNA genome and prevented NS5A from interacting with lipid droplets, cellular lipid storage organelles that are required for assembly of new viruses. We propose that domain I of NS5A plays multiple roles in virus assembly. As domain I is the putative target for one class of DAAs, our observations may have implications for the as yet undefined mode of action of these compounds.

## Introduction

Hepatitis C virus (HCV) is a member of the Flaviviridae family of enveloped, positive-strand RNA viruses [1]. It is estimated to infect up to 170 million individuals globally [2]. HCV causes inflammation and fibrosis in the liver via damage to hepatocytes. Over time, chronic infection progresses to significant fibrosis and may lead to cirrhosis with a risk for decompensation and hepatocellular carcinoma (HCC) [3].

The HCV genome is approximately 9,600 nucleotides in length and comprises 5’ and 3’ untranslated regions (UTRs) flanking a single open reading frame encoding a 3,000-residue polyprotein precursor [4,5]. Co- and post-translational proteolytic cleavage of this precursor by cellular and viral enzymes yields the structural proteins: Core, envelope glycoproteins E1 and E2, and the p7 ion channel, which are involved in viral assembly, along with non-structural (NS) proteins NS2, NS3, NS4A, NS4B, NS5A and NS5B. With the exception of NS2, which is dispensable for RNA replication and may control virus assembly, the other 5 NS proteins (NS3-NS5B) are necessary and sufficient for membrane-associated RNA replication [6]. By definition, NS proteins are expressed in virus-infected cells but are not incorporated into virus particles; although directly involved in RNA synthesis, they also play roles in modulation of host defence mechanisms and virus assembly [7,8]. In addition to NS5A, whose roles are detailed below, recent studies have provided evidence for the involvement of NS3, NS4B and NS5B in the later stages of the virus lifecycle–namely virus assembly and release [9–13].

Over the past few years there have been extraordinary advances in the therapy for HCV infection–the standard IFN and ribavirin therapy has been rapidly superseded by combination therapy with a range of direct-acting antivirals (DAAs) targeting the NS3/4A protease, NS5A, and the NS5B RNA-dependent RNA polymerase. As one important target of DAAs, NS5A is a ~450 amino acid multi-functional phosphoprotein that has essential roles throughout the virus life cycle. It is composed of three domains (I, II and III) linked by low complexity sequences (S1A Fig), although in recent years domains II and III have been increasingly defined as a single, unstructured domain. The protein is anchored to phospholipid membranes by an N-terminal amphipathic helix (residues 1–33) in a manner essential for replication [14]. The structure of domain I has been solved by three independent groups using X-ray crystallography. These studies revealed four different dimeric forms of domain I from genotype 1a and 1b with the same monomeric unit, but different dimeric arrangements [15–17]. By primary sequence comparison, domain I of NS5A shares a high sequence homology among all hepaciviruses, while domain II and III exhibit a lower level of homology [18–22]. These observations suggest that domain I has critical and well conserved functions that are common to all hepaciviruses, whereas the functions of the other two domains may be specific to individual viruses. In this regard, it is generally accepted that the function(s) of domain I are required exclusively for genome replication [23], many culture-adaptive mutations map to this domain, and the majority of domain II together with all of domain III are dispensable for replication [24–26].

In HCV infected cells, NS5A localizes to the endoplasmic reticulum (ER), virus–induced multiple-membrane vesicles (MMV) that host RNA replication complexes (also called the membranous web), and to lipid droplets. The MMV contain the NS proteins NS3-NS5B and virus RNA and represent sites of active genome replication [27–30]. The precise role of NS5A in genome replication remains obscure, however it is widely accepted that this is mediated by binding to viral RNA [31,32], other NS proteins and interactions with various cellular factors, including vesicle-associated membrane protein-associated proteins A and B (VAP-A, VAP-B), cyclophilin A (CypA) and phosphatidylinositol-4-kinase IIIα (PI4KIIIα), which are required for HCV replication [33–37].

Following RNA replication, nascent viral genomes need to be transported from the sites of RNA replication to distinct, as yet poorly characterised, sites of virus assembly. Here infectious virus particles are generated, bringing together the structural proteins and the viral genome to be packaged in a temporally and spatially organized manner [8,38]. An increasing body of evidence points to a role of NS5A in coordinating this process, possibly by transporting the genome RNA to assembly sites and delivering it to the Core protein for encapsidation. A further level of complexity arises from the fact that, compared to other enveloped positive-strand viruses, a key feature of infectious HCV particles is that they exhibit unusually low buoyant densities, while particles with higher buoyant densities are less infectious [39–43]. Indeed highly purified HCV particles are rich in lipids and cholesterol resembling very-low density lipoproteins (VLDL) [44,45]. This property requires that cellular lipid droplets (LDs), lipid storage organelles surrounded by a phospholipid monolayer, are involved in HCV assembly.

Both Core and NS5A are targeted to lipid droplets, and this recruitment is essential for virus assembly. Mutations that block either Core or NS5A localization to LDs inhibit virus production, suggesting that LDs are intimately involved in virus particle assembly [46–48]. The function of NS5A in virus assembly has been mapped to domain III. Mutations close to the C-terminus of domain III disrupt the ability of NS5A to interact with Core, abrogate infectious particle formation and lead to an enhanced accumulation of Core on the surface of LDs [49]. In addition, a number of cellular NS5A-interacting partners have been implicated in LD function/targeting and virus assembly. These include Apolipoprotein E (ApoE), diacylglycerol acyltransferase-1 (DGAT-1), Annexin A2 and Rab18 [50–55]. Of note, both DGAT-1 and Rab18 have been reported to recruit NS5A on to LDs and are proposed to play roles in transporting NS5A (and most likely genome RNA) between replication sites and LDs/assembly sites [52,55]. Although virus encapsidation could occur at the LD, it is noteworthy that LDs are only surrounded by a phospholipid monolayer, therefore the virions cannot obtain their lipid envelope from them. Assembly of an infectious enveloped HCV virion particle must ultimately require that Core and virion RNA are transported from LDs [29] to a membranous compartment, possibly involving the ESCRT and/or endosomal pathways [56–58].

In this study, we present evidence that domain I of NS5A also plays a key role in the assembly of infectious virus. We identify two key surface exposed, conserved residues that, when substituted with alanine, retain genome replicative capacity but block the production of infectious virus. We show that these mutations inhibit the ability of HCV to perturb LD structure and distribution and disrupt the recruitment of NS5A to LDs. They also impair the dimerization of domain I and enhance the binding of domain I to the HCV 3’UTR RNA, revealing a role for these NS5A attributes in virus assembly.

## Results

### Generation of a panel of alanine substitutions in domain I

In comparison with domain II and domain III, domain I of NS5A is highly conserved throughout all HCV isolates, and is also well conserved in related viruses such as GB virus type B (GBV-B) and the novel hepaciviruses that have recently been identified in a variety of species (S1B Fig). In addition, the structure of domain I has been determined by three independent groups [15–17]–all three studies agree on the monomer structure but show these monomers assembling into dimers with different monomer orientations and dimer interfaces (S1C Fig). In this study we initially set out to define residues in domain I that were required for viral genome replication. To this end, we first aligned amino acid sequences from 29 isolates representing all 7 HCV genotypes, together with 10 related viruses such as bat hepacivirus (BHV), GB virus-B (GBV-B), guereza hepacivirus (GHV), non-primate hepacivirus (NPHV) and rodent hepacivirus (RHV) (S1 Table). This analysis revealed 24 absolutely conserved residues (S2 Table). We then mapped these conserved residues on to the two genotype 1b structures (PDB 1ZH1 and 3FQM) of domain I to identify surface exposed residues, particularly those that are charged. This analysis identified 11 residues that were then targeted for alanine scanning mutagenesis and subsequent profiling in the context of the JFH-1 sub-genomic replicon (SGR) and infectious virus. In addition, a conserved surface exposed cluster (residues 153 to 158) was mutated collectively to alanine as these residues were located in close proximity on the tertiary structure (S2 Table).

### Role of domain I in RNA replication

To investigate the role of the selected conserved residues in domain I, the mutants were cloned into a previously described JFH-1 derived SGR (mSGR-luc-JFH-1) [25] in which the NS5A coding sequence was flanked by unique restriction sites generated by mutagenesis to facilitate sub-cloning. Importantly, these modifications did not alter the coding capacity of the polyprotein and had no effect on replication of the SGR [25]. RNAs transcribed from the mutant panel were electroporated into Huh7 cells and luciferase activity was measured at 4, 24, 48 and 72 h post electroporation (h.p.e.). The luciferase activity at 4 h.p.e. correlates with translation of input transcripts prior to onset of replication and subsequent time points were normalized to the 4 h.p.e. signal to account for electroporation efficiency. As a negative control an inactive mutant of the NS5B polymerase was used (GND) [59].

**Fig 1:**
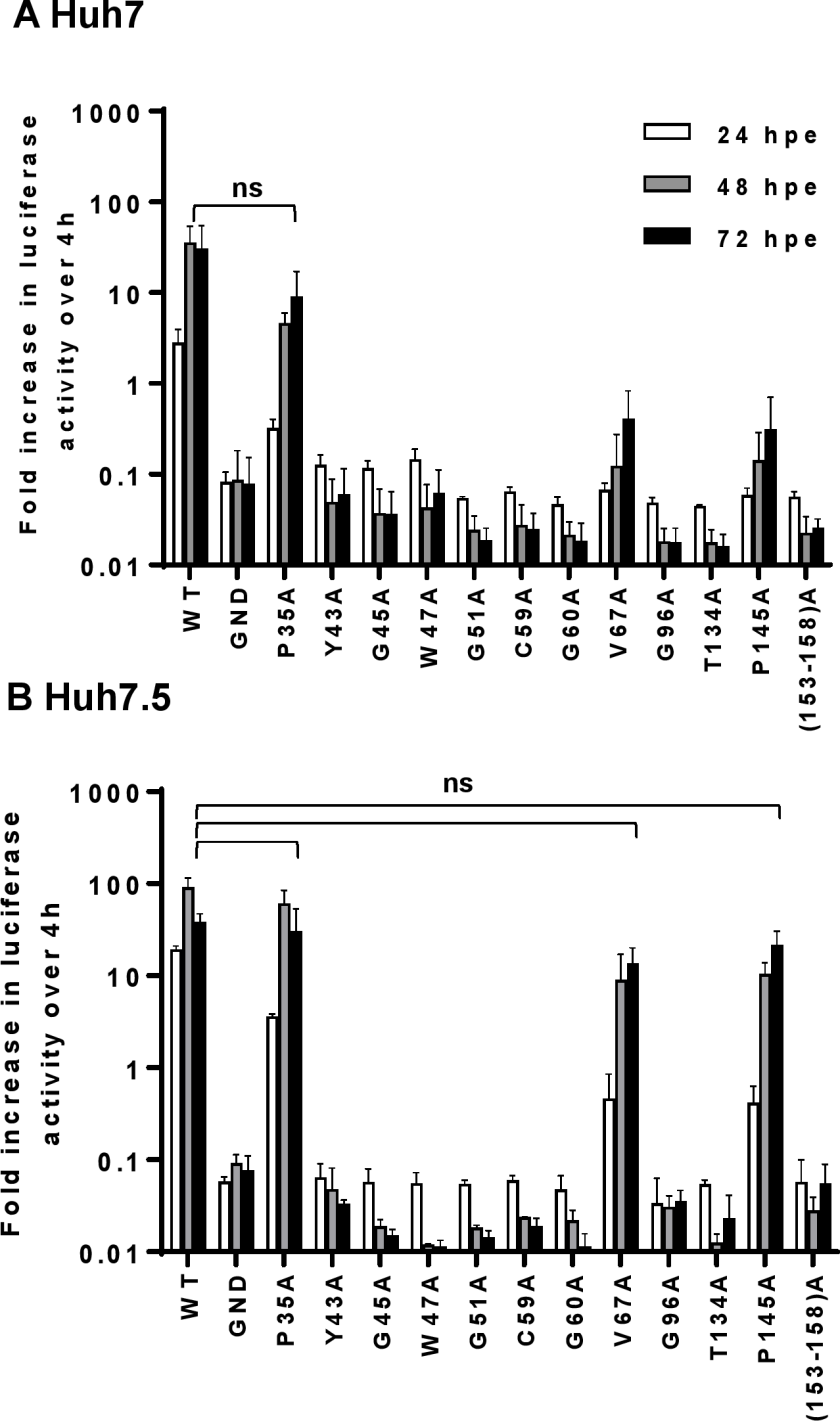
Genome replication phenotypes of NS5A domain I mutants in Huh7 and Huh7.5 cells. *In vitro* transcripts of mSGR-luc-JFH-1 containing the indicated mutations were electroporated into either Huh7 (**A**) or Huh7.5 (**B**) cells. Luciferase activity was measured at 4, 24, 48 and 72 h post-electroporation (h.p.e.) and was normalized to 4 h.p.e. Data from three independent experiments are shown and error bars represent the standard error of the mean. ns: no statistically significant difference from WT.

Nine of the mutations (Y43A, G45A, W47A, G51A, C59A, G60A, G96A, T134A and 153-158A) were shown to completely disrupt the ability of the mSGR-luc-JFH-1 to replicate in Huh7 cells (Fig 1A), being indistinguishable from the GND negative control. However, three mutants (P35A, V67A and P145A) were able to replicate, albeit at levels lower than wild type (WT). P35A exhibited a modest but non-significant defect, in contrast V67A and P145A replicated at significantly lower levels than WT (p<0.05) (Fig 1A). All mutants showed broadly comparable luciferase activity at 4 h.p.e., demonstrating that the replication phenotypes observed were not due to differences in electroporation efficiency (S2 Fig).

We then assessed whether the replication defects exhibited by these mutants could be due to the low permissibility of Huh7 cells for HCV replication, rather than a lack of replicative capacity. To test this we evaluated the mutation panel in Huh7.5 cells which were derived from Huh7 cells, and are highly permissive for HCV genome replication [60]. As shown in Fig 1B, those mutants that were unable to replicate in Huh7 cells (Y43A, G45A, W47A, G51A, C59A, G60A, G96A, T134A and 153-158A) exhibited the same phenotype in Huh7.5 cells, confirming that these residues are absolutely required for the function of NS5A in genome replication. However, the three mutants that were able to replicate in Huh7 cells, albeit at a lower level than WT, (P35A, V67A and P145A) were able to replicate more efficiently in Huh7.5 cells, reaching levels almost equivalent to the WT with modest but non-significant impairment **(**Fig 1B). However, it was important to confirm that this permissiveness in Huh7.5 cells was not a phenomenon that was specific for domain I. To this end, an SGR containing a mutation (D329A) within NS5A domain II [61], which we previously reported replicated approximately 5-fold lower than WT, was electroporated into both Huh7 and Huh7.5 cells. As shown in S3A Fig, D329A was also able to replicate more efficiently in Huh7.5, demonstrating that this effect was not specific for domain I.

We proceeded to confirm that the replication phenotypes observed resulted from the loss (or disruption) of a specific function of NS5A, rather than a defect at the level of polyprotein translation or proteolytic processing. To this end, all 12 mutations were cloned into a plasmid in which the expression of the NS3-5B proteins of JFH-1 was driven by the human cytomegalovirus (CMV) promoter (pCMV10-NS3-5B), thus allowing replication–independent expression of these replicase proteins (S3B Fig). These plasmids were transfected into Huh7.5 cells and cell lysates were analysed for protein expression by western blot at 48 h post transfection (hpt), using HCV NS3 as a polyprotein processing control. All 12 mutants expressed levels of NS5A and NS3 comparable to WT (p ≥0.1) (S3B Fig). This confirmed that the replication phenotypes of these mutants were not the result of effects on NS5A translation, stability and/or polyprotein cleavage.

### A novel role for domain I in virus assembly

**Fig 2:**
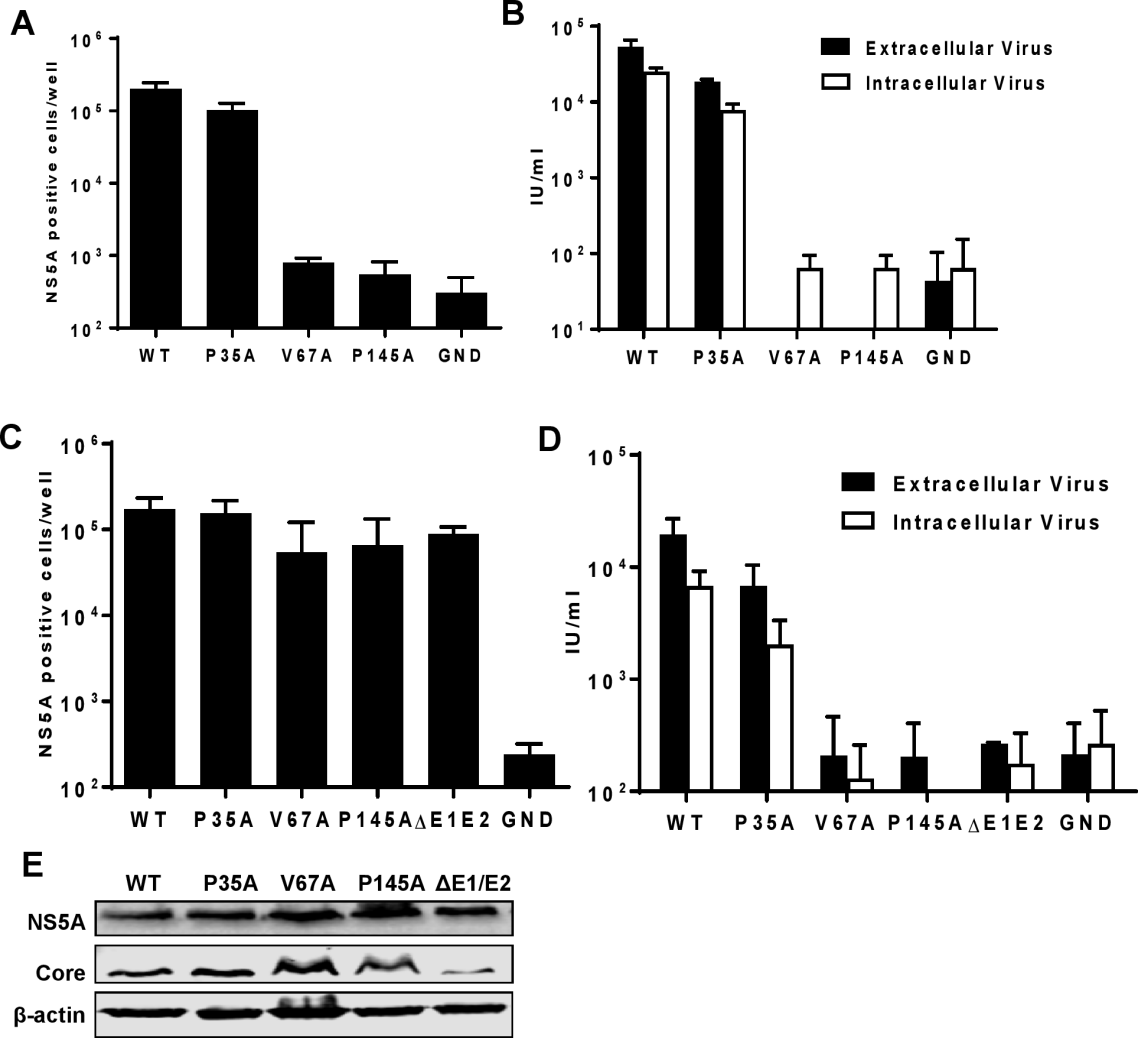
Mutations in NS5A domain I disrupt the production of infectious virus. *In vitro* transcripts of mJFH-1 containing the indicated mutations were electroporated into either Huh7 (**A, B**), or Huh7.5 (**C-E**) cells. Virus genome replication and protein expression was assayed by quantification of NS5A positive cells 48 h.p.e. for Huh7 (**A**) or Huh7.5 (**C**) cells by using the Incucyte-ZOOM [62]. (**B**, **D**) Intracellular and extracellular infectious virus was titrated at 72 h.p.e. **E** Huh7.5 cell lysates at 72 h.p.e. were analysed by western blot with anti-NS5A, anti-Core and anti-β-actin antibodies. Data from three independent experiments are shown and error bars represent the standard error of the mean.

To determine whether the attenuation of genome replication for P35A, V67A and P145A in Huh7 cells was also observed in the context of infectious virus, these mutations were sub-cloned into the full-length mJFH-1 infectious clone. This construct contains the same unique restriction sites flanking NS5A as mSGR-luc-JFH-1, and the nucleotide sequence changes did not affect the levels of virus assembly and release [25] Following electroporation of full-length virus transcripts into Huh7 cells we determined virus genome replication activity by quantification of the number of NS5A positive cells using the IncuCyte ZOOM at 48 h.p.e. as previously described [62]. As expected, replication of P35A, V67A and P145A in the context of infectious virus (Fig 2A) was consistent with the observation in SGRs (Fig 1A). P35A exhibited a modest reduction which was not significant, whereas V67A and P145A showed a ~100-fold reduction in replication and were indistinguishable from the GND negative control. Consistent with this replication phenotype, neither V67A nor P145A produced any infectious virus particles, either within the cells (intracellular virus), or released into the supernatant (extracellular virus) (Fig 2B). A different picture emerged when these mutant virus RNAs were electroporated into Huh7.5 cells. As shown in Fig 2C, replication of P35A was indistinguishable from WT, whereas both V67A and P145A showed only a modest defect. This result was confirmed by western blot analysis for NS5A and Core expression (Fig 2E). However, despite the restoration of genome replication to WT levels, V67A and P145A were unable to produce any infectious virus (Fig 2D). This phenotype mirrored that of the additional control used in this experiment, ΔE1-E2 (a deletion within the envelope glycoprotein coding region previously shown to be unable to assemble infectious virus)[25,49]. As noted previously [62], although the IncuCyte ZOOM allows for rapid automated quantification of virus titres, the sensitivity of the instrument does result in a high background. However, visual inspection of samples (for example see S4 Fig) confirmed the absence of infectivity for V67A, P145A and negative controls.

We conclude from these data that the two residues V67 and P145 are partially required for genome replication, as mutations of these residues resulted in a reduction of replication that could be rescued by the increased permissibility of Huh7.5 cells. In contrast these two residues are absolutely required for the assembly of infectious HCV particles. This result was surprising, as it is widely accepted that domain I of NS5A is exclusively involved in genome replication. The one exception to this is the report 10 years ago showing that alanine scanning mutagenesis of residues 99–101 or 102–104 had no effect on genome replication, but blocked release of infectious virus from Huh7.5 cells [44], although whether these mutants affected assembly of intracellular infectious virus was not determined. We reasoned that the ability of V67A and P145A to replicate to near WT levels in Huh7.5 cells offered the opportunity to assess the role of domain I in virus assembly, without any confounding replication defect that would make interpretation of the data difficult.

However, before analysing the phenotype of V67A and P145A in more detail, we confirmed that the phenotypes of these mutants were not due to the acquisition of an additional compensatory mutation during the cloning process. To do this, we generated revertant viruses in which the WT NS5A coding sequence was sub-cloned back into the V67A and P145A virus backbones. As shown in S5A Fig, following electroporation of revertant RNA into Huh7.5 cells, both genome replication and production of both intracellular and extracellular virus was restored to WT levels.

**Fig 3:**
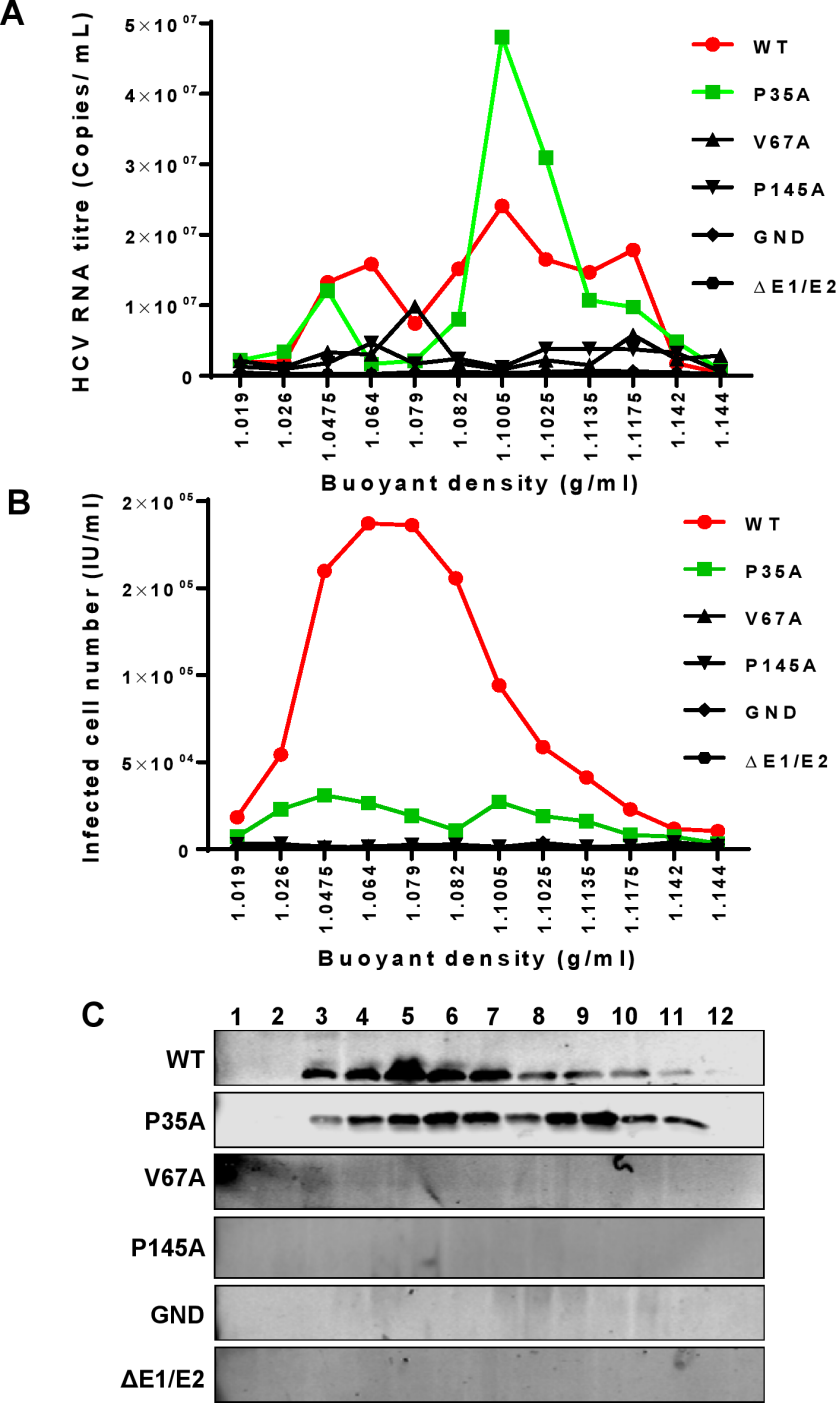
Density gradient analysis of mutant viruses. Huh7.5 cells were electroporated with *in vitro* transcripts of WT or the indicated virus mutants. Concentrated culture medium was fractionated using 10–40% iodixanol density-gradient centrifugation. For each fraction, HCV RNA (**A**) and infectivity (**B**) were plotted against the buoyant density (n = 3), and Core protein in each fraction was detected by western blot (C). 1 to 12 in (C) indicated the fractions collected from top to bottom with the buoyant density indicated in (A) and (**B**). The result of a representative of three independent experiments is shown.

We considered that the failure of V67A and P145A to produce infectious virus was either due to a gross assembly defect such that no virus particles were generated, or that virus particles were assembled but were non-infectious. Such non-infectious particles might be empty, lacking the genome, or could exhibit some other more subtle defect such as a failure to associate with lipids. To test this hypothesis, culture medium from Huh7.5 cells electroporated with JFH-1 WT, P35A, V67A and P145A RNA was concentrated and fractionated by iodixanol density-gradient centrifugation. As controls, cells were electroporated with GND and ΔE1/E2 RNAs. Each fraction was analysed by quantitative RT-PCR (Fig 3A) to determine the presence of genomic RNA, and infectivity was measured using the Incucyte ZOOM as described [62] (Fig 3B). As expected JFH-1 WT showed a broad peak of infectivity at a low density (1.064 g/ml) that coincided with a genomic RNA peak, a second larger RNA peak at a higher density (1.1005 g/ml) was less infectious, consistent with previous reports [44]. P35A also showed two coincident peaks of infectivity and RNA, although the majority of the viral RNA was associated with the higher density fraction which exhibited less infectivity. In contrast, no genomic RNA or infectivity could be detected for either V67A or P145A, these two mutants were indistinguishable from the two negative controls (GND and ΔE1/E2). Gradient fractions were concentrated by methanol precipitation prior to analysis for the presence of Core by western blot. This analysis (Fig 3C) revealed a complete lack of any Core protein in fractions from either V67A or P145A, again in common with the negative controls. In contrast both WT and P35A exhibited Core protein correlating with the peaks of infectivity and virus RNA. We conclude that both V67A and P145A mutations block the assembly of infectious virus particles at an early stage. Of note, unlike the replicase function of domain I [63], the assembly function was unable to be trans-complemented by wildtype NS5A: following co-electroporation of V67A or P145A mutant JFH-1 RNA with a wildtype SGR no infectious virus was produced (S5B Fig). This is consistent with a recent study revealing that the assembly function of NS5A domain III was refractory to trans-complementation [64].

### A role for NS5A domain I in the redistribution and formation of lipid droplets during infection

**Fig 4:**
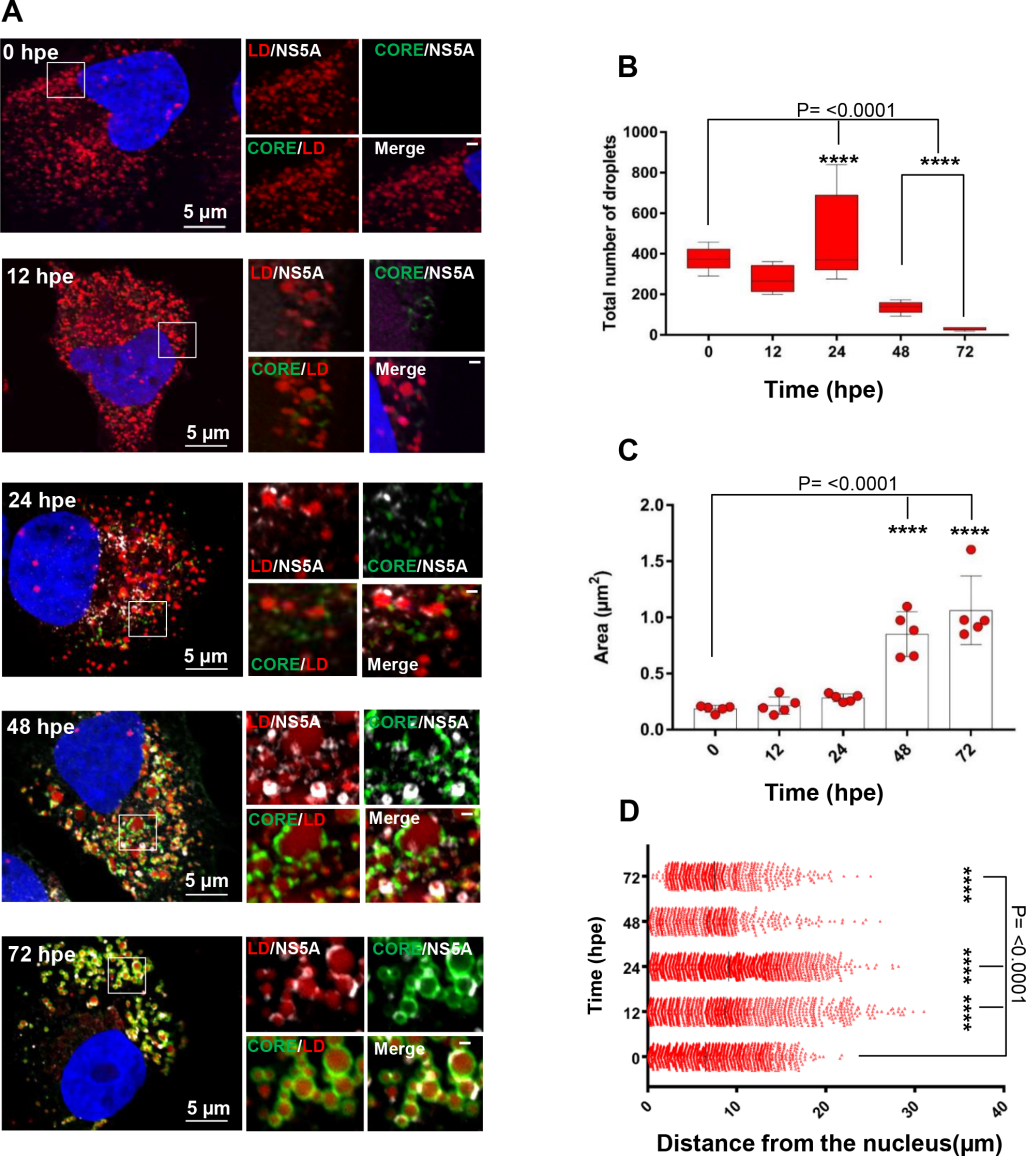
Time-course immunofluorescence analysis of LDs, NS5A and Core in WT infected cells. Huh7.5 cells were electroporated with an *in vitro* transcript of mJFH-1 WT. At the indicated h.p.e. cells were fixed and stained with anti-NS5A and Core antibodies, BODIPY 558/568-C_12,_ and DAPI and imaged by Airyscan microscopy (**A**). Spatial data for LDs were determined from 10 cells for each time point using Fuji. These data were used to determine the number of LDs per cell (**B**), the average size of LDs (**C**) and the distance of each LD from nucleus at different time points (**D**). **** indicates significant difference (P<0.0001) from the results for LDs in untransfected cells. The scale bars are 5μm and 0.5 μm, respectively.

To shed light on the phenotype of the V67A and P145A mutations, we applied an imaging approach, using high resolution confocal microscopy (Airyscan) to assess the distribution of both viral and cellular factors during infection [65,66]. In this regard, lipid droplets (LD) are important organelles for the assembly of infectious HCV particles, although their precise role remains to be elucidated [44]. Both Core and NS5A have been shown to localise with LDs and infection with HCV results in dramatic changes to the distribution and size of LDs. This is demonstrated in Fig 4: Huh7.5 cells were electroporated with JFH-1 WT RNA and analysed by Airyscan confocal microscopy for the distribution of LD, Core and NS5A at various time-points up to 72 h.p.e. (Fig 4A). The number (Fig 4B), and total area of LDs (Fig 4C), together with their distance from the nuclear membrane (Fig 4D), were determined. During the first 12 h the number of LDs declined slightly, but then increased at 24 h, followed by a further dramatic decline by 48/72 h. Importantly however, the total area of LDs within the cytoplasm (a measure of the amount of lipids stored in LDs) increased significantly at 48/72 h, indicative of an increase in the size of LDs. There were more subtle changes to the distribution of LDs: at early times (12/24 h)—they scattered throughout the cytoplasm, whereas later the distribution was more restricted to the perinuclear area (48 h) and exhibited a clustering (72 h). As previously documented, both Core and NS5A were associated with LDs at later time points. Core can be seen to completely coat the surface of LDs whereas NS5A is restricted to punctate areas on the surface. We observed the same pattern of changes in cells infected with JFH-1 (S6A Fig).

**Fig 5:**
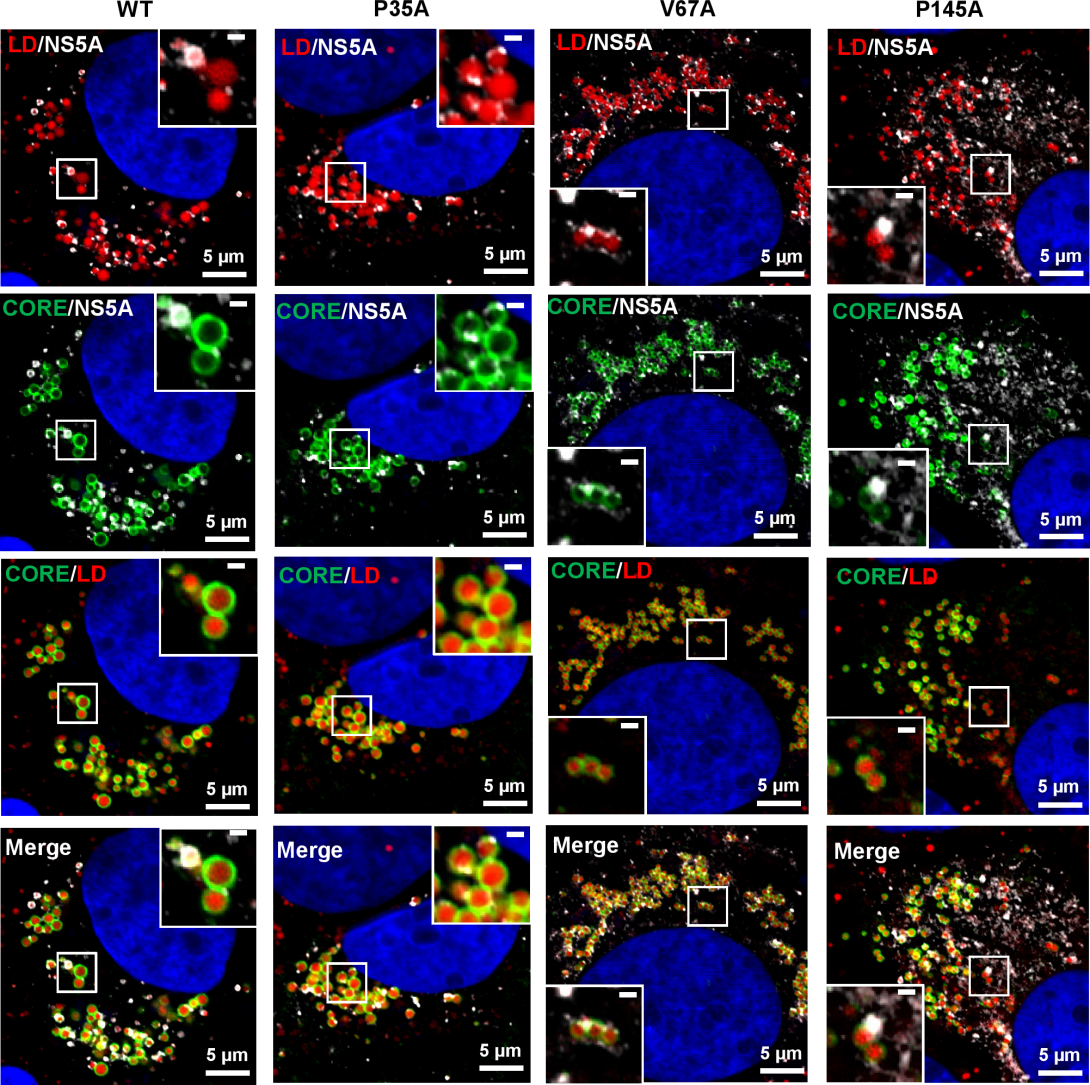
Subcellular distribution of Core and NS5A relative to the LDs in infected cells is disrupted by domain I mutations V67A and P145A. Spatial distribution of Core and NS5A relative to the LD in Huh7.5 cells electroporated with *in vitro* transcripts of either wild-type mJFH-1, or NS5A mutants P35A, V67A and P145A. Cells were seeded onto coverslips and incubated for 72 h.p.e. prior to fixation and immunostaining for Core (rabbit, 1:500), NS5A (sheep, 1:2000) and LD (BODIPY 558/568-C_12,_ 1:1000), and imaging by Airyscan microscopy. The scale bars are 5μm and 0.5 μm, respectively.

**Fig 6:**
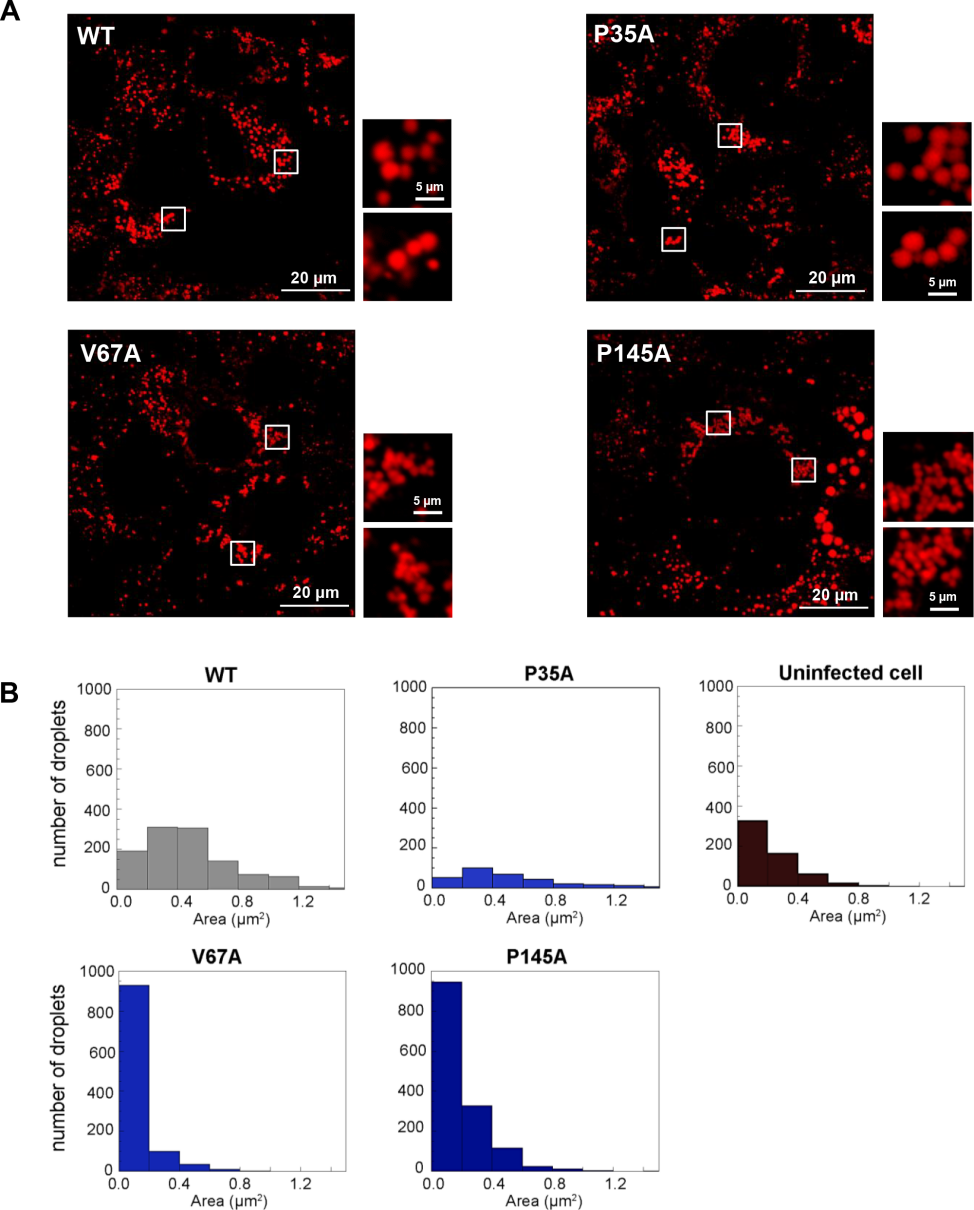
Quantification of the effect of the V67A and P145A mutations on the size of LD. **A** LDs in Huh7.5 cells electroporated with the indicated JFH-1 constructs were visualized by staining with BODIPY 558/568-C_12_. **B** The size of individual LD was determined and plotted as a histogram. The area (μm^2^) is taken as an indication of the three-dimensional volume of the LD. For comparison similar data was determined from uninfected Huh7.5 cells.

**Fig 7:**
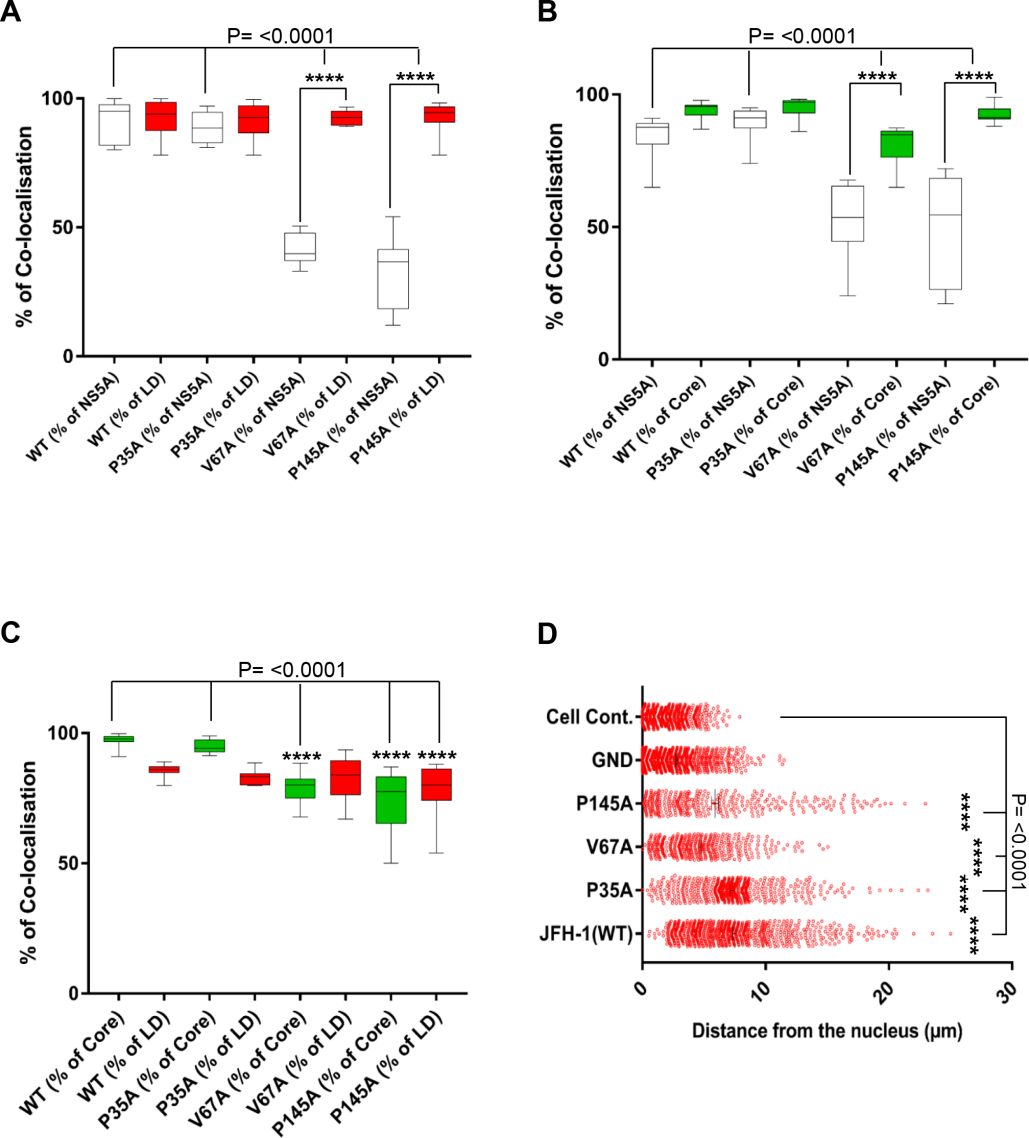
V67A and P145A disrupted the co-localization between NS5A and Core or LDs. **A** Quantification of the percentages of NS5A colocalized with LD (white blocks), or LD colocalised with NS5A (red blocks). **B** Quantification of the percentages of NS5A colocalized with Core (white blocks), or Core colocalised with NS5A (green blocks). **C** Quantification of the percentages of Core colocalized with LD (green blocks), or LD colocalised with Core (red blocks). **D** Spatial data for the distance of LDs from the nuclear envelope were determined from 10 cells for each construct using Fiji. **** indicates significant difference (P<0.0001) from the results for WT.

We then examined the distribution of LDs, Core and NS5A at 72 h.p.e. in Huh7.5 cells electroporated with RNA for the three domain I mutants, P35A, V67A and P145A (Fig 5). Airyscan imaging of these cells revealed some striking differences: P35A was largely indistinguishable from WT but V67A and P145A exhibited distinct phenotypes. The most notable difference was that for V67A and P145A the size of the LDs was dramatically reduced compared to WT and P35A. Quantification confirmed this visual conclusion (Fig 6A), in WT and P35A infected cells the majority of LDs had an area of between 0.2–0.6 μm^2^, whereas for V67A and P145A infected cells, and uninfected controls, the majority were below 0.2 μm^2^ (Fig 6B). In addition, there were some other differences between WT/P35A and V67A/P145A: in particular the amount of NS5A localised at the surface of lipid droplets appears to be much less for the latter two mutants. This was confirmed by quantitative analysis (Fig 7A), the percentage of NS5A fluorescence that co-localised with LD was significantly reduced. However the reciprocal analysis (percentage of LD that co-localised with NS5A) showed no differences. This suggested that the proportion of LDs that were associated with NS5A was no different to WT. However, compared to WT, the majority of NS5A did not associate with LDs. Quantitative analysis of the NS5A:Core co-localisation revealed a similar trend whereby the percentage of NS5A co-localised with Core was significantly less for V67A and P145A (Fig 7B). In contrast, although the percentage of Core that co-localised with LD was significantly reduced for V67A and P145A, the reduction was much less dramatic (Fig 7C). Lastly, we observed that there were differences in the distribution of LDs: for both V67A and P145A the LDs were significantly closer to the nucleus, albeit not as close as in either GND-electroporated or mock control cells (Fig 7D).

**Fig 8:**
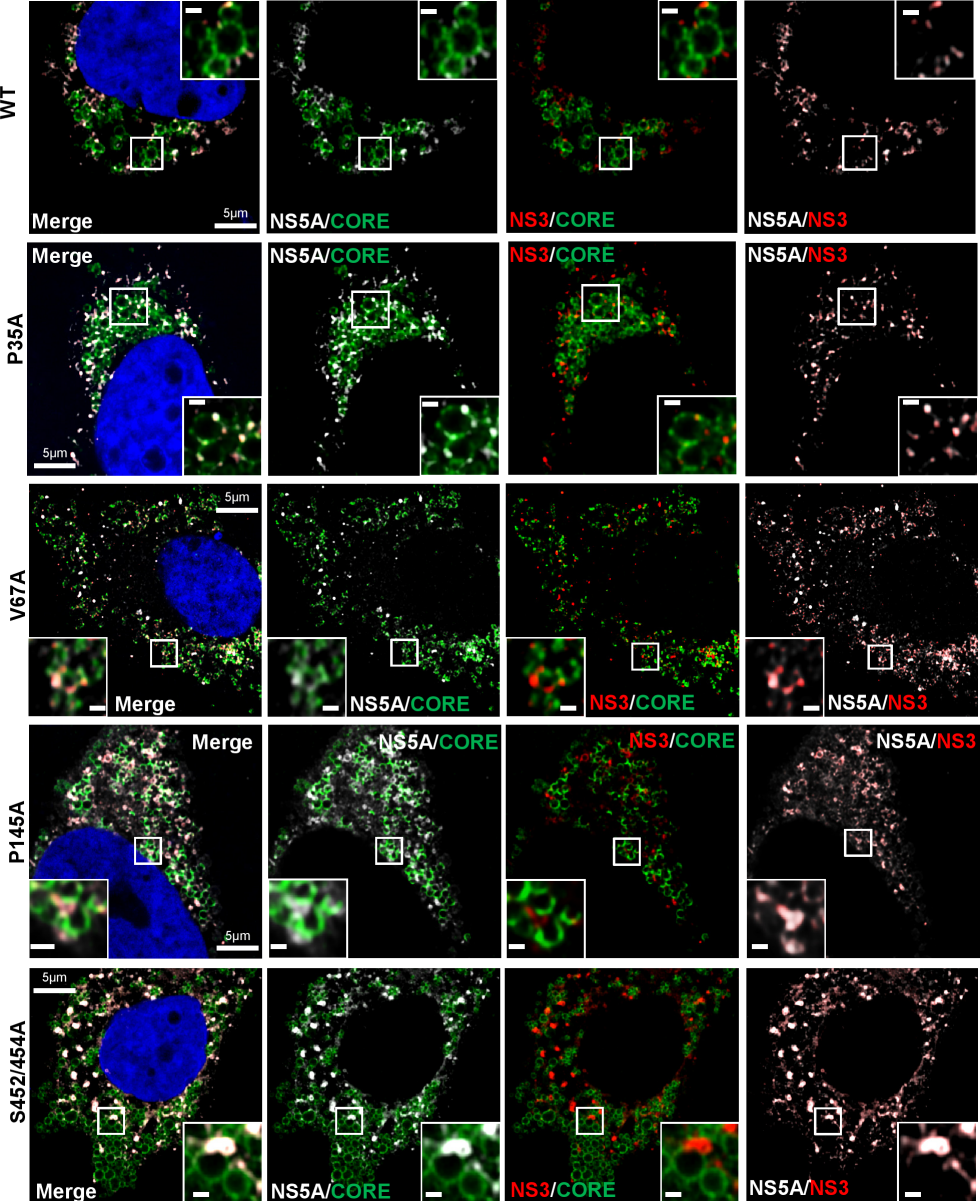
Co-localisation of NS5A, Core and NS3 in infected cells. Huh7.5 cells were electroporated with *in vitro* transcripts of mJFH-1 WT or the indicated mutants. At 72 h.p.e. cells were fixed and stained with anti-NS5A, NS3 and Core antibodies, and counterstained with DAPI, prior to imaging by Airyscan microscopy. The scale bars are 5 μm and 0.5 μm, respectively.

As the colocalisation of NS5A with Core and LDs was reduced for V67A and P145A, we also investigated the colocalisation with another replicase component, NS3. This analysis revealed a high level of colocalisation of NS5A and NS3 (Fig 8), In this analysis we also included a mutant within domain III of NS5A (S452A/454A), previously shown by us to exhibit a 100-fold reduction in production of infectious virus [25]. Interestingly, this showed a distinct phenotype with large puncta positive for both NS5A and NS3, and LDs comparable to WT/P35A. Quantification (S6B Fig) revealed that in fact V67A and P145A exhibited a modest but significant reduction in NS5A:NS3 colocalisation, suggesting that these mutations disrupt the interactions between NS5A and both the assembly machinery (Core and LDs), but also to a lesser extent the replicase components.

**Fig 9:**
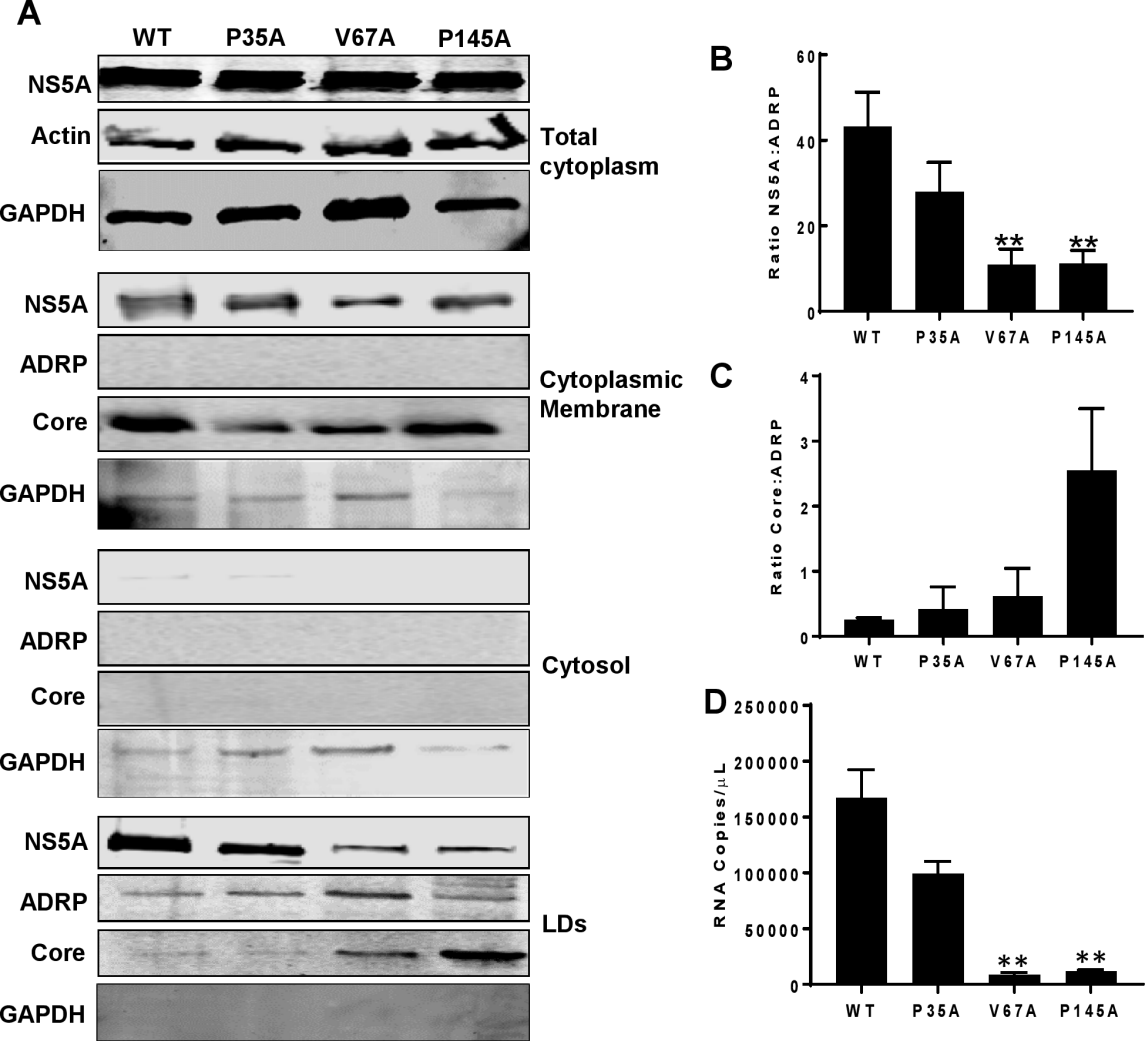
V67A and P145A disrupt the recruitment of NS5A and Core to LDs. **A** Western blot analysis of NS5A and Core proteins, the LD marker protein ADRP and GAPDH in purified LD fractions compared with whole cytoplasm, cytoplasmic membrane and cytosolic fractions. The abundance of NS5A (**B**) and Core (**C**) in the LD fractions was quantified and normalised to the LD fraction ADRP value. **D** Amount of viral RNA in LD fractions was determined by qRT-PCR. Error bars represent the standard error of the mean of three independent experiments. ** indicates significant difference (P<0.01) from WT.

We complemented this imaging analysis by investigating the biochemical composition of LDs. LDs were purified from electroporated cells by density gradient centrifugation and analysed by western blot for NS5A and Core, using antibody to the LD-associated adipose differentiation-related protein (ADRP, also known as adipophilin or perilipin 2) [44,67] as a marker for LDs. The integrity of the LDs and lack of contamination with other cellular components was demonstrated by the absence of GADPH [68]. As shown in Fig 9, ADRP was exclusively present in the LD fraction (not in the cytosolic or membrane fractions). Both NS5A and Core were also detected in the LD fractions, however the relative distribution and amounts of these two viral proteins differed between the mutants and WT. Both V67A and P145A showed significantly less NS5A in the LD fraction (Fig 9B), consistent with the fluorescence data (Figs 5 and 7A). In contrast the amount of Core in the LD fraction of V67A and P145A was increased (Fig 9C). We also used qRT-PCR to quantify the amount of viral RNA in the LD fractions. This analysis revealed that for both V67A and P145A there was a significant reduction in genomic RNA associated with LDs (Fig 9D), consistent with a scenario whereby NS5A transports nascent genomes to LDs where it is transferred to the Core protein for subsequent movement to assembly sites.

### V67 and P145 modulate RNA binding and domain I dimerization

**Fig 10:**
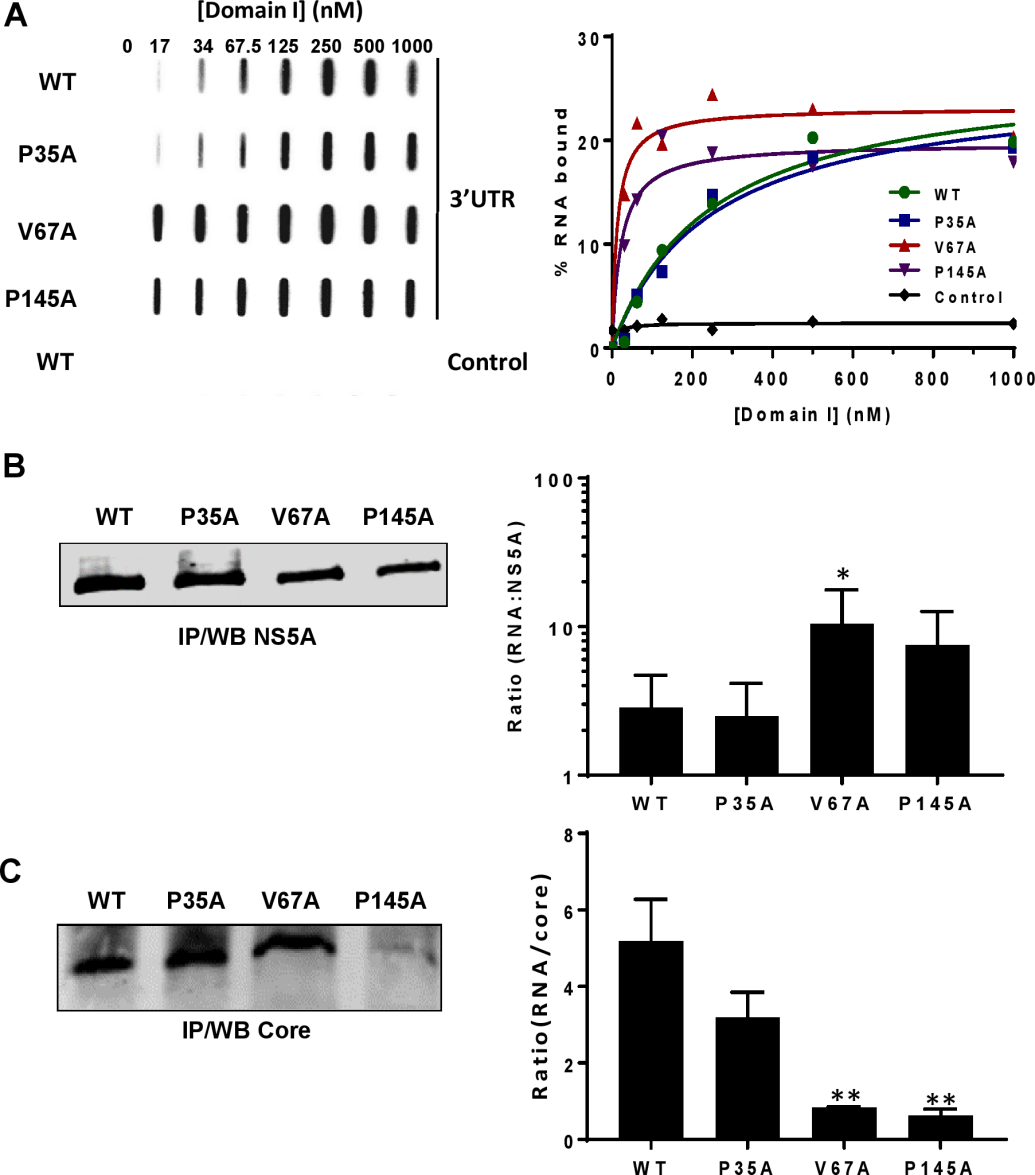
Residues at positions V67 and P145 of domain I are involved in NS5A RNA binding. **A** Representative slot blot analysis of RNA-protein complexes captured on nitrocellulose membrane in a filter binding assay using increased amounts of purified His-tagged NS5A domain I (S6 Fig), and a constant amount of ^32^P-labelled HCV 3’UTR (or control RNA [32]). % RNA bound is shown graphically, quantified by phosphoimaging analysis. **B** Huh7.5 cells were electroporated with *in vitro* transcripts of mJFH-1 WT or the indicated mutants. Cells were lysed at 72 h.p.e. and NS5A was immunoprecipitated from cell lysates. After washing the beads were subjected to analysis by Western blot and RNA extraction. qRT-PCR were performed to quantify the level of (+) genome RNA bound to NS5A. The graph on the right shows the ratio of RNA copies to NS5A (n = 2). **C** As **B** but in this case Core was immunoprecipitated using a rabbit polyclonal anti-Core antibody. ** indicates significant difference (P<0.01) from WT.

Implicit in the above scenario is the specific interaction of NS5A with genomic RNA. In this context, domain I has been shown by us, and others [31,32,69], to bind specifically to the HCV 3’UTR RNA. We therefore asked whether the three mutations affected this binding capacity. To address this, we expressed domain I WT and the three mutants as His-SUMO fusion proteins in *E*.*coli*. The fusion proteins were purified and cleaved to release the untagged domain I (S7 Fig). The RNA binding capacity of the WT and mutant domain I proteins was determined by RNA filter binding assay utilizing ^32^P-labelled HCV 3’UTR RNA (Fig 10A). Surprisingly, we found that V67A and P145A showed strong binding affinity to HCV 3’UTR RNA, exhibiting a 10–20 fold increase compared to WT or P35A. For WT and P35A the Kd values were 246.3 ± 77.19 nM and 245.7 ± 70.09 nM respectively. However for V67A and P145A, the values were 12.89 ± 6.25 nM and 22.35 ± 9.58 nM respectively.

To validate this *in vitro* data, we immunoprecipitated NS5A from Huh7.5 cells electroporated with either JFH-1 WT or the three mutants and assessed the amount of viral RNA in the immunoprecipitates by qRT-PCR. Consistent with the *in vitro* RNA filter binding assay data, both V67A and P145A bound more viral RNA compared to WT and P35A (Fig 10B). In contrast, a similar analysis of Core immunoprecipitates revealed significant reductions in the amount of genomic RNA bound to Core for V67A and P145A (Fig 10C). Taken together, these data suggest that NS5A binds specifically to the nascent genomic RNA but that during the assembly process this must be released to Core. By increasing the affinity of NS5A for the 3’UTR RNA, these mutations are preventing this transfer.

**Fig 11:**
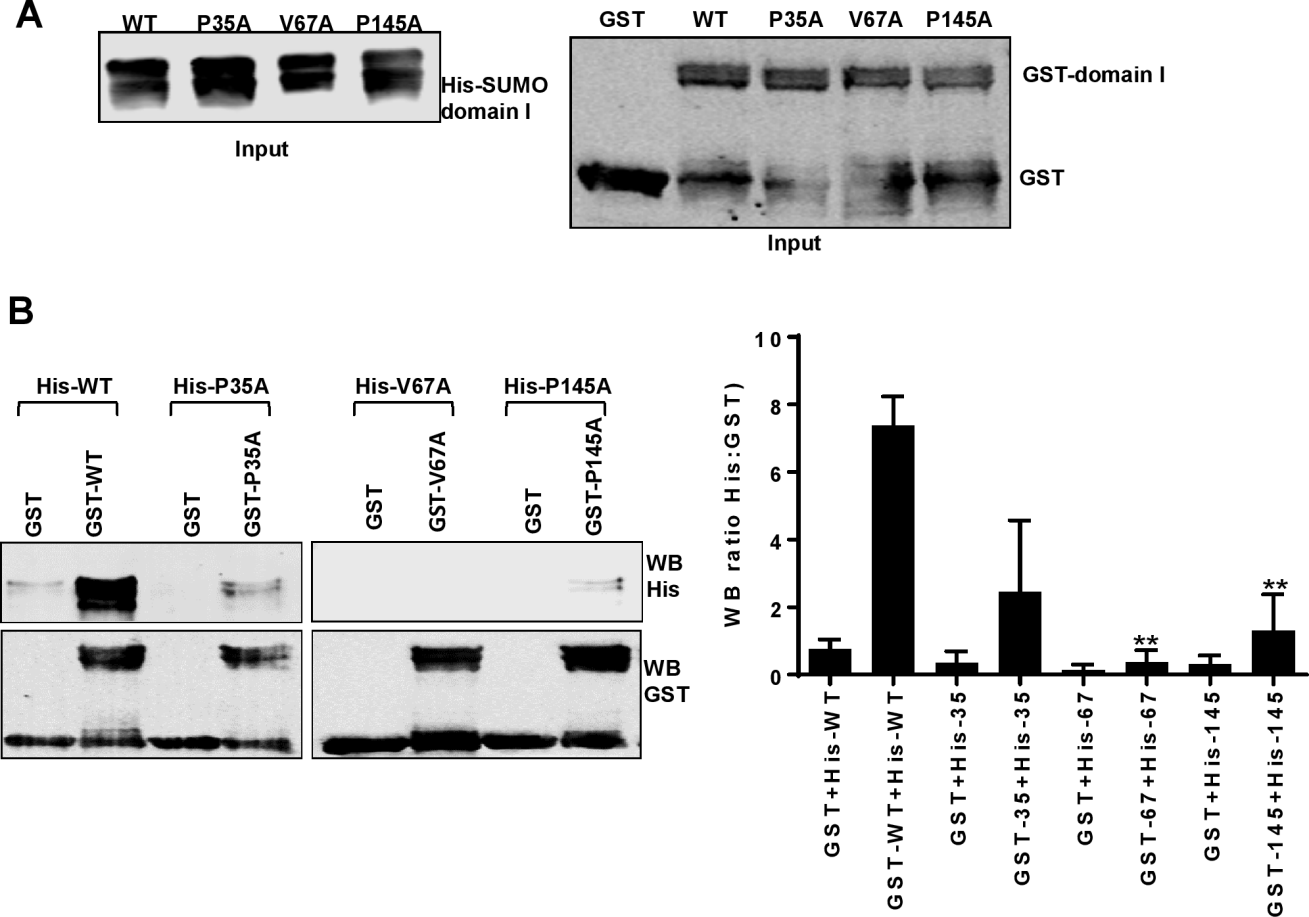
Residues at positions V67 and P145 of domain I are involved in NS5A dimerization. **A** Input of His-SUMO-domain I (35–215) (left), GST control protein and GST-domain I (35–215) (right), analysed by Western blotting using either anti-His or anti-GST antibodies. **B** His-tagged domain I proteins were also used as prey in pulldown assays with GST or GST-Domain I with corresponding mutations as bait. Precipitated proteins were analysed by Western blotting using anti-His and anti-GST antibodies. The His:GST ratio was calculated following quantification of Western blot signals using a Li-Cor Odyssey Sa infrared imaging system and represented graphically as a measure of the dimerization activity. These data were representative of three independent experiments using different batches of purified domain I proteins. ** indicates significant difference (P<0.01) from WT.

NS5A has also been reported to dimerize, both in the published crystal structures [15–17] and in biochemical analyses [70]. Examination of the different dimer structures revealed that P35 was located in the dimer interface of the ‘open’ conformation [15,71,72]. P145 was located in the interface of the ‘closed’ conformation [15–17,72]. In contrast V67 was distal to the dimer interfaces in both conformations (S8 Fig). To test the effects of the three mutations on dimerization, we conducted GST pulldown assays using GST-tagged domain I as bait to precipitate His-tagged domain I (input levels of proteins shown in Fig 11A). We observed that GST-domain I (WT), but not GST alone, precipitated His-domain I (WT) (Fig 11B). GST-domain I (P35A) was also able to precipitate His-domain I (P35A) with a modest but non-significant reduction in binding. In contrast, both V67A and P145A mutant GST-domain I proteins failed to precipitate the cognate His-domain I proteins (Fig 11B), indicating that these two residues are required for dimerization of domain I and implicating a role for NS5A dimerization in virus assembly.

## Discussion

This study identified three residues in NS5A domain I for which alanine substitution had a modest effect on genome replication, but significant defects in the assembly of infectious virus particles. These residues were chosen for their conservation–P35 and P145 are 100% conserved throughout all hepaciviruses, V67 is conserved in all HCV genotypes apart from genotype 4 where it is generally an isoleucine. Structural analyses of domain I also predicted that they are all surface exposed. In particular we focussed our attention on two of these, V67A and P145A, which completely abrogated virus assembly. Previously, domain I has been assumed only to function during genome replication, and to our knowledge this is the first detailed analysis of a role for domain I in virus assembly.

Both V67A and P145A mutants failed to produce intracellular infectious virus and consequently failed to release any virus particles, as judged by the lack of virus RNA or Core protein in cell culture supernatants. This was not due to a lack of genome replication or Core protein within the cells, as levels of both were similar to WT (S4 Fig and Fig 2). In cells infected with V67A or P145A mutant viruses there were defects in LD production. Compared to WT, LD were smaller, closer to the nucleus and NS5A recruitment to LDs was impaired. Lastly, these two mutants enhanced binding of domain I to the HCV 3’UTR RNA and inhibited dimerization.

What are the implications of these data? Firstly, they imply that domain I of NS5A plays multiple roles in virus assembly. It is required both for the association of NS5A with LD as well as the increase in LD size and altered distribution (movement away from the nuclear membrane) that is seen during HCV infection. Taken together with the *in vitro* data, these support a model in which domain I of NS5A binds to the 3’UTR of nascent genomes and transports them from sites of replication to LD. Here, analogous to the handing on of a baton in a relay race, the RNA is transferred to Core and then subsequently transported to assembly sites. The latter remain to be unambiguously defined but may be endosomal membrane compartments [73,74]. The enhanced binding of V67A or P145A to the 3’UTR RNA may prevent the release of RNA for transfer to Core. The LD distribution in cells infected with V67A or P145A at 72 h.p.e. ressembles that in wildtype at 12/24 h.p.e., suggesting that these mutations might block the transition from genome replication to virus assembly. Furthermore, the loss of dimerization by these two mutants implies that, in contrast to the accepted model of an open NS5A dimer revealing a basic RNA-binding groove, monomeric NS5A is able to bind RNA. However, we cannot rule out the possibility that in the intact protein, domains II and III influence both dimerization and RNA binding by domain I. In this regard we note that our attempts to detect NS5A dimerization within intact cells have so far been unsuccessful, despite testing a variety of experimental protocols (see S9A Fig). Despite this, it is tempting to speculate that monomeric NS5A might transport nascent RNA to LDs, then dimerizes and releases the RNA to Core.

Our data are consistent with previous studies into the role of NS5A during virus assembly which support a model whereby NS5A orchestrates the processes of genome replication and virus assembly. However, these studies have exclusively focused on the role of domain III [49], and it has been widely accepted that the determinants of virus assembly within NS5A lie entirely within domain III. For example, a serine near the C-terminus of domain III is implicated in the interaction between NS5A and Core, and it has been proposed that phosphorylation of this residue by casein kinase II is required for virus assembly [75]. More recently, mutations of a basic cluster at the N-terminus of domain III resulted in modest impairment of Core-RNA and NS5A-RNA interactions and virus particle envelopment, leading to a 100-fold reduction in released virus titres [76]. Our data extend these observations, providing evidence that domain I also makes a major contribution to virus assembly.

Other implications of our study concern the modifications to LD morphology that occur during HCV infection. As illustrated in Fig 4, at late stages (48 h onwards), increases in LD size and total volume most likely reflect the coalescence of smaller LDs into larger structures. Our data indicate that domain I of NS5A plays a role in this process, as V67A and P145A do not exhibit this increase (Figs 5 and 6). NS5A is recruited to LDs, in most cases to discrete punctate locations on the surface, in contrast to the complete coating of LDs with Core.

One apparent discrepancy in our data relates to the co-localisation of Core with LDs. Specifically, the imaging data (Figs 5 and 7) showed a modest reduction in Core:LD co-localisation for V67A and P145A, whereas these mutants showed higher levels of Core co-purified with LDs (Fig 9). Two factors may help to explain this discrepancy: firstly, it is possible that in the case of V67A and P145A, Core associates more strongly with LDs, possibly because it has not been displaced by NS5A. Secondly, V67A and P145A infected cells exhibit larger numbers of smaller LDs, thus the available LD surface area for interaction with Core is also likely to be larger, allowing more Core to associate. In addition, it is important to note that the data in Fig 7C refer to the percentage of total Core associated with LD, and do not take into account the absolute amounts of Core.

Whether the increase in LD size is a direct consequence of recruitment of NS5A, or indirectly driven by NS5A-mediated effects on lipid metabolism, remains unclear. In this context, NS5A has previously been shown to interact with a number of LD-associated proteins, including DGAT-1 [77] and Rab18 [78]. However, the phenotype of V67A or P145A cannot be explained by a lack of binding to these proteins–as shown in S9B Fig, both DGAT-1 and (to a lesser extent) Rab18 precipitated with both WT and the three mutant NS5As. We are currently extending this analysis, using a proteomic approach to determine the interactome of the three mutant NS5As in comparison to WT.

In contrast to V67A and P145A, P35A exhibited a moderate virus assembly phenotype with only a small (less than 10-fold) reduction in virus titre. Nevertheless some important observations can be made: firstly, in the density gradient analysis (Fig 3) the peak of infectivity for P35A resolved at a lower buoyant density than WT (1.0475 g/ml compared with 1.064 g/ml). In contrast the second peak of infectivity with higher buoyant density for P35A was associated with more genome RNA and Core than WT. These data imply subtle differences in the association of virus particles with VLDL or other lipids. In all other analyses (LD size and distribution, NS5A recruitment to LD, dimerization and 3’UTR binding), P35A was not statistically significantly different from WT.

Lastly, it is important to consider our results in the context of the class of potent DAAs that are defined as NS5A inhibitors, exemplified by daclatasvir (DCV). Although initially developed as inhibitors of genome replication [79], it has become clear that DCV also has an independent effect on virus assembly. Treatment of infected cells with DCV resulted in a rapid (2 h) block to virus assembly, preceding the inhibition of genome replication which was only apparent at later time points (24 h) [80]. More recently, it has been shown that DCV treatment prevented the transfer of genomic RNA to assembly sites [81]. DCV has been reported to target domain I, as judged by the location of DCV-resistance mutations (eg L31M and Y93H). It is important to note that none of the 3 mutations analysed in this study exhibited any effect on the activity of DCV measured against HCV genome replication (S10 Fig). However, our observation that domain I is directly implicated in virus assembly does provide a rationale for the rapid effect of DCV on this process, and may therefore help to explain the extraordinary potency of DCV and related compounds.

## Materials and methods

### Plasmids

DNA constructs of luciferase reporter sub-genomic replicon (mSGR-luc-JFH-1), infectious mJFH-1 virus and sub-genomic replicon with NS5A containing the One-Strep-tag (OST) (pSGR-Neo-JFH1-5A-OST) were maintained in our laboratory [82]. pcDNA3.1(+) was used as the vector to subclone the *Bam*HI-*Hin*dIII JFH-1 NS5A fragment for site-directed mutagenesis. NS5A fragments with mutations were then cloned into either mSGR-luc-JFH-1 or mJFH-1 via flanking *Bam*HI/*Afe*I restriction sites. The pCMV10-NS3-5B plasmid was constructed [61], and the NS5A domain I fragments with mutations were then inserted into this wild type vector by cloning the *Nsi*I–*Rsr*II fragment containing the mutations from the corresponding mJFH-1 constructs. NS5A-OST with mutations from pSGR-Neo-JFH1-5A-OST were cloned back into mJFH1 viruses via N*si*I and B*sr*GI restriction sites to generate mJFH1-5A-OST constructs. Primer sequences available upon request.

### Antibodies

The following antibodies were used: sheep anti-NS5A (in house polyclonal antiserum) [83], mouse anti-NS5A (9E10) (kind gift from Tim Tellinghuisen, Scripps Florida), mouse anti-NS3 (kind gift from Thomas Pietschmann, TWINCORE, Hannover), rabbit anti-Core (polyclonal serum R4210) and sheep anti-ADRP (kind gifts from John McLauchlan, Centre for Virus Research, Glasgow), sheep anti-GST (in-house), mouse anti-DGAT1 (Santa Cruz), mouse anti-Rab18, anti-Actin and anti-His (Sigma Aldrich).

### Luciferase-based sub-genomic replicon assay

Huh7 and Huh7.5 cells that are highly permissive for HCV RNA replication were used for electroporation [60]. Cells were washed twice in cold phosphate-buffered saline (PBS) before electroporating 4x10^6^ cells in cold PBS with 2 μg of RNA at 975 μF and 260 V. Cells were resuspended in complete media before being seeded into either 96-well plates (n = 6) at 3x10^4^ cells/well, or 6-well plates (n = 2) at 3x10^5^ cells/well, both plates incubated under cell culture conditions. 4, 24, 48 and 72 h post-electroporation (h.p.e.), cells were harvested by lysis with 30 μl or 200 μl passive lysis buffer (PLB; Promega) from 96- and 6-well respectively. Luciferase activity was determined from 96-well samples on a BMG plate reader by automated addition of 50 μl luciferase assay reagent (Promega) and total light emission was monitored.

### Western blot analysis

Cells were washed twice with PBS, lysed by resuspension in Glasgow lysis buffer (GLB) [1% Triton X-100, 120 mM KCl, 30 mM NaCl, 5 mM MgCl2, 10% glycerol (v/v), and 10 mM piperazine-N,N’-bis (2-ethanesulfonic acid) (PIPES)-NaOH, pH 7.2] supplemented with protease inhibitors and phosphatase inhibitors (Roche Diagnostics), and incubated on ice for 15 min. Following separation by SDS-PAGE, proteins were transferred to a polyvinylidene fluoride (PVDF) membrane and blocked in 50% (v/v) Odyssey blocking buffer (LiCor) in Tris-buffered saline (TBS) [50 mM Tris, 150 mM NaCl, pH 7.4]. The membrane was incubated with primary antibody in 25% (v/v) Odyssey blocking buffer overnight at 4°C, then incubated with fluorescently labelled anti-sheep (800nm), anti-rabbit (800nm) or anti-mouse (700 nm) secondary antibodies for 2 h at room temperature (RT) before imaging on a LiCor Odyssey Sa fluorescent imager.

### Virus replication and titration

Huh7.5 cells were washed twice in cold PBS before electroporating 2x10^7^ cells in cold PBS with 10μg viral RNA at 975 μF and 260 V. Cells were resuspended in complete medium and seeded into 6-well plates and T175 flasks for virus replication and virus titration analysis.

48 h.p.e., cells were washed in PBS and fixed in 4% paraformaldehyde (PFA) for 20 min and staining with NS5A-specific sheep polyclonal antiserum as primary antibody (dilution 1:2000) and Alexa Fluor-594 conjugated donkey anti-sheep (Invitrogen) as a secondary antibody (dilution 1:750) for IncuCyte counting (see details in **Use of the Incucyte ZOOM**).

Culture supernatants in T175 flasks were harvested at 72 h.p.e., and extracellular virus titres were determined. Intracellular infectivity was determined for freeze–thaw lysates of electroporated cells 72 h.p.e. using the protocol reported previously [84]. Naïve Huh-7.5 cells were seeded into 96 well plates (8.0x10^3^ cells/well, 100 μL total volume) and allowed to adhere for 6 h. Clarified virus was serially diluted two-fold into the existing media (final volume 100 μL per well). Cells were incubated for 48h post infection (hpi) before the detection of viral antigens by indirect immunofluorescence. Virus-positive cells were counted using IncuCyte and the titre (IU/mL) was calculated from the wells of multiple virus dilutions [31].

### Use of the IncuCyte ZOOM

Following immunofluorescence staining for viral antigens, with an Alexa Fluor 594-conjugated (“red”) secondary antibody, fixed microtitre plates were imaged with the IncuCyte ZOOM (Essen BioScience) [62] to determine the total number of virus-positive cells/well. Viral titres were obtained by multiplying the number of virus-positive cells/well by the reciprocal of the corresponding dilution factor, corrected for input volume. As this method measures the absolute number of infected cells, rather than the number of foci of infected cells, the titre is represented as infectious units per mL (IU/mL).

### Purification of HCV particles

Culture medium from JFH-1 infected cells was concentrated 100-fold using 10% PEG 8000 (w/v) (Fisher Scientific) and centrifugation at 3000 g for 30 min. The pellet was resuspended in 1ml of PBS and overlaid over a 1 ml cushion (20% sucrose, w/v, in PBS), followed by ultracentrifugation at 150,000 g for 3 h at 4°C in an S55S rotor. The resulting pellet was resuspended in 200 μl PBS and then loaded on a 10–40% gradient iodixanol in 2.2 mL tubes followed by centrifugation at 150,000 g for 4 h at 4°C. The gradient was fractionated into 12 fractions of 180 μl each. Each fraction was used for virus titration as well as RNA extraction for qRT-RCR analysis, the remainder of each fraction was mixed with ice-cold methanol (1:3) and proteins precipitated at -80°C overnight. Precipitated proteins were recovered by centrifugation at 13,000 rpm for 30 min at 4°C, and pellets were resuspended in 25 μl SDS-PAGE loading buffer, prior to western blot analysis.

### Quantitation of HCV RNA by qRT-PCR

To quantify the number of HCV genomes, RNA from each fraction after gradient centrifugation of extracellular virus was extracted using TRIzol following the manufacturer’s instructions (Invitrogen). Extracted cellular RNA was analysed by qRT-PCR using a one-step qRT-PCR Taqman-based kit as directed by the manufacturer (Eurogentec). Amplifications were conducted in triplicate using the following primers and 6FAM- and TAMRA- labelled probes designed to detect the HCV JFH-1 5’UTR: 5’UTR Taqman probe 83–108: 5’- 6FAM-CATGGCGTTAGTATGAGTGTCGTACA-TAMRA-3’; 5’UTR Forward-57: 5’-CTGTCTTCACGCAGAAAGCG-3’; 5’UTR Reverse-312: 5’-CACTCGCAAGCGCCCTATCA-3’.

### Immunofluorescence analysis

Virus RNA electroporated cells were seeded onto 19 mm glass coverslips in 12 well plates, 72 h.p.e. cells were fixed in 4% PFA and permeabilised with 0.1% (v/v) Triton X-100 (Sigma-Aldrich) in PBS for 7 min. Coverslips were washed twice in PBS and the primary antibody applied at the relevant dilution in 10% (v/v) FBS in PBS and incubated for 2 h at RT. To remove any unbound primary antibody, cells were washed three times in PBS before the application of the relevant Alexa Fluor-488, 594 or 647 conjugated secondary antibodies diluted 1:750 in 10% (v/v) FBS in PBS followed by 2 h incubation at RT in the dark. Lipid droplets were stained using BODIPY (558/568)-C_12_ dye at 1:1000 (Life Technology). The coverslips were washed three times in PBS before the nucleus was stained by the addition of 4’,6’-diamidino-2-phenylindole dihydrochloride (DAPI) diluted 1:10 000 in PBS for 30 min at RT in the dark. Coverslips were washed three times in PBS and mounted on a glass microscope slide in ProLong Gold antifade regents (Invitrogen, Molecular Probes) and sealed with nail varnish. Slides were stored at 4°C in the dark until required and examined. Confocal microscopy images were acquired on a Zeiss LSM880 microscope with Airyscan, post-acquisition analysis was conducted using Zen software (Zen version 2015 black edition 2.3, Zeiss) or Fiji (v1.49) software [85].

### Co-localisation analysis

For co-localisation analysis, Manders' overlap coefficient was calculated using Fuji ImageJ software with Just Another Co-localisation Plugin (JACoP) (National Institutes of Health) [73]. Coefficient M1 reports the fraction of the LD signal that overlaps either the anti-NS5A or anti-Core signal or the fraction of anti-Core signal that overlaps the anti-NS5A signal. Coefficient M2 reports the fraction of either the anti-NS5A or anti-Core signal that overlaps the LD signal or the fraction of anti-NS5A that overlaps the anti-Core signal. Coefficient values range from 0 to 1, corresponding to non-overlapping images and 100% co-localization images, respectively. Co-localisation calculations were performed on >10 cells from at least two independent experiments.

### Quantification of LD distribution and size

For the quantification of LD spatial arrangement, images were acquired with the same acquisition parameters, but with variable gain to ensure correct exposure. The two-dimensional coordinates of the centroids of LDs were calculated using the Analyze Particles module of Fiji (ImageJ). The distance of each particle to the edge of the nucleus, visualised using DAPI stain, was looked up using a Euclidean distance map computed with the Distance Transform module of Fiji and exported as a list of distance measurements via the Analyze Particle function. Box and whisker plots of these distance measurements were constructed using GraphPad Prism and compared between samples using a one-way ANOVA and Bonferroni-corrected post-hoc t-tests. Two-dimensional areas of the LDs were also measured using the Analyze Particles function in Fiji. Lists of the area measurements were used for constructing frequency histograms using a custom-written programme implemented in IDL. The shapes of these histograms were compared using a chi-squared test, implemented in IDL.

### Isolation of lipid droplets

Four 10 cm dishes of Huh7.5 cells electroporated with mJFH-1 virus RNA (80% confluent) were scraped into 10 mL of PBS at 72 h.p.e.. The cells were pelleted by centrifugation at 1,500 rpm for 5 min and then resuspended with 500 μL buffer A (20mM Tricine, 250mM sucrose, pH 7.8) supplemented with protease and phosphatase inhibitors and kept on ice for 20 min. The suspension was homogenized with a plastic tissue grinder homogenizer. Samples after homogenization were centrifuged at 3000g for 10 min at 4°C to remove nuclei and the post nuclear supernatant (PNS) was collected, transferred into 2.2 mL tubes and overlaid with 1 mL of buffer B (20 mM HEPES, 100 mM KCl and 2 mM MgCl_2_ pH 7.4) plus protease inhibitors. Tubes were centrifuged in a S55S rotor at 100,000g for 1h at 4°C. After centrifugation, the LD fraction on the top of the gradient was recovered in buffer B and washed twice by centrifugation at 20,000g for 5 min at 4°C to separate the LDs from the buffer. Underlying solution was removed and discarded. Proteins and lipids in LD samples were separated with 2 volumes of ice-cold acetone and chloroform (1:1) to precipitate proteins. RNA in lipid droplet fractions were extracted using TRIzol for qRT-PCR. The collected LD fraction was dissolved in 50μL of SDS sample loading buffer for western blot.

### GST-pulldown assay

Construction and purification of domain I with corresponding mutations have been listed in **S1 Text.** After purification, GST-domain I (GST-DI) and His-SUMO-domain I (His-SUMO-DI) were dialyzed against dialysis buffer (50 mM Tris-HCl, pH 7.5, 100 mM NaCl, 5 mM MgCl_2_, 10% glycerol, 0.5% NP-40). A GST pulldown assay was performed as described previously [70]. Briefly, 10 μg of GST or GST-fusion proteins were mixed with 5 μg of His-SUMO-DI in binding buffer (20mM Tris-HCl, pH 7.2, 0.5 M NaCl, 200KCl, and 1% NP-40) for 3 h at 4°C on a rotating platform. Then the mixture was added to glutathione beads and incubated overnight at 4°C. After washes using binding buffer, bound material was eluted with 50 μL of SDS sample buffer and heated for 10 min at 95°C. After centrifugation, these samples were analysed by Western blot using anti-GST and anti-His antibodies.

### RNA filter binding assay

His-SUMO-DI proteins were cleaved with SUMO protease to produce native domain I. Following purification as in **S1 Text,** domain I was incubated with *in vitro* transcribed [α-^32^P] radiolabelled RNAs as described previously [32]. Then aliquots of each binding reaction were applied to a pre-assembled slot blot apparatus and filtered through firstly a nitrocellulose membrane (Schleicher & Schuell) to capture soluble protein-RNA complexes, and secondly a Hybond-N nylon membrane (Amersham Biosciences) to bind free RNA. After washing and air drying of both membranes, quantification of radioactivity was performed by phosphoimaging using an FLA 5000 Imaging system (Fuji), and ImageJ software. These data were fitted to the hyperbolic equation $R=R_{max}\times P/(Kd+P)$. R is the percentage of bound RNA, R_max_ is the maximal percentage of RNA competent for binding, P is the concentration of Domain I, and Kd is the dissociation constant [32].

### Co-immunoprecipitation of Core or NS5A and viral RNA

Co-immunoprecipitation experiments were performed in Huh7.5 cells 72 h.p.e. with mJFH-1 virus RNA using polyclonal anti-Core or monoclonal anti-NS5A antibodies and Dynabeads™ Protein G (Thermo Fisher Scientific), following the manufacturers protocol. Immunoprecipitated proteins were subjected to immunoblotting and co-immunoprecipitated RNA was extracted by TRIzol reagent and then quantified by qRT-PCR.

### Statistical analysis

Statistical analysis was performed using unpaired two-tailed Student’s *t* tests, unequal variance to determine statistically significant differences from the results for the wild type (n≥3). Data in histograms are displayed as the means ± S.E.

## Supporting information

S1 Fig Structure and conservation of NS5A. **A. Schematic representation of the domain organization of NS5A.** The three domains (I-III), the linking low complexity sequences (LCSI and II), and the membrane anchoring amphipathic helix (AH) are illustrated. Numbers indicate positions of amino acids in the JFH-1 genotype 2a NS5A sequence. **B. Conservation of three different NS5A domains from HCV isolates representing each genotype and related hepaciviruses.** Isolates used for analysis are listed in S2 Table. Filled bars in different colours indicate the percentage conservation at each residue as indicated in the key below. Gaps refer to locations where there are insertions in the JFH-1 sequence, compared to consensus, particularly the 18 amino acid insertion between residues 432–450. **C. Analysis of the three dimensional structures of domain I (1ZH1 and 3FQM) using Pymol.** Residues highlighted are the conserved amino acids that are located on the surface of two dimeric conformations at positions indicated in S1 Table.
(TIF)

S2 Fig Genome replication of NS5A domain I mutants. *In vitro* transcripts of mSGR-luc-JFH-1 containing the indicated mutations were electroporated into either Huh7 (**A**) or Huh7.5 (**B**) cells. Luciferase activity was measured at 4, 24, 48 and 72 h post-electroporation (h.p.e.) and plotted as absolute values. 4 h.p.e. values are indicative of input translation and reflect transfection efficiency. Data from three independent experiments are shown and error bars represent the standard error of the mean.
(TIF)

S3 Fig Comparison of replication of NS5A mutants in Huh7 and Huh7.5 cells and analysis of polyprotein processing. **A**. WT represents the wild type mSGR-luc-JFH-1. P35A, V67A, and P145A are the mutants of domain I which can replicate at lower levels than WT in Huh7 cells; D329 is located at the C terminus of NS5A domain II. The graph shows the RLU values at 72 h.p.e. expressed as a fold increase over the 4 h.p.e. values. **B.** Huh7.5 cells were transfected with pCMV10-NS3-NS5B expression vectors containing the corresponding mutations. At 48 h.p.t., cell lysates were harvested in GLB and analysed by SDS-PAGE and Western blotting with anti-NS5A (sheep) and anti-NS3 (mouse). The ratio of NS5A:NS3 was calculated following quantification of Western blot signals using a Li-Cor Odyssey Sa infrared imaging system. Data from three independent experiments are shown and error bars represent the standard error of the mean.
(TIF)

S4 Fig Incucyte ZOOM visualisation of virus replication and infection. Indirect immunofluorescence analysis for NS5A expression in Huh7.5 cells electroporated with the indicated viral RNAs at 48 h.p.e. (top row). The middle row shows NS5A expression in cells infected with culture supernatants harvested from the cells presented in the top row. Infected cells were analysed at 48 h.p.i. The bottom row shows NS5A expression at 48 h.p.i. in cells infected with cell lysates from the cells in the top row–this represents intracellular virus. After fixation, cells were stained with NS5A antibody and then with Alexa Fluor 568-conjugated donkey anti-sheep IgG (red fluorescence).
(TIF)

S5 Fig Revertant and trans-complementation analysis of the phenotype of V67A and P145A in virus assembly. **A.** Phenotypes of V67A and P145A are not derived from acquisition of an additional compensatory mutation during the cloning process. Revertants were generated by cloning a WT NS5A fragment back into the mJFH-1 V67A or P145A mutant plasmids. Huh7.5 cells were electroporated with in vitro transcripts of the resulting V67 or P145 revertants. Virus genome replication and protein expression was assayed by quantification of NS5A positive cells 48 h.p.e. by using the Incucyte-ZOOM [62]. Intracellular and extracellular infectious virus was titrated at 72 h.p.e. **B.** In vitro transcribed WT JFH-1 or the indicated mutant RNAs were co-electroporated with the helper RNA (mSGR-Luc-JFH1) into Huh7.5 cells. 72 h.p.e., supernatant was harvested and cells were lysed by repetitive freeze-thaw cycles. Extracellular and intracellular virus was then titrated in Huh7.5 cells and viral infectivity was determined by using Incucyte ZOOM at 48h.p.i. Data from two independent experiments are shown and error bars represent the standard error of the mean.
(TIF)

S6 Fig **A. Time-course immunofluorescence analysis of LD in HCV infected cells.** Huh7 cells were infected with mJFH-1 WT at an M.O.I. of 0.5 ffu/cell. At the indicated h.p.e. cells were fixed and stained with BODIPY 558/568-C_12,_ and DAPI and imaged by Airyscan microscopy. **B. Colocalisation of NS5A and NS3.** Quantification of the percentages of NS5A colocalized with NS3 (white blocks), or NS3 colocalised with NS5A (red blocks) as shown in Fig 8. Co-localisation calculations were performed on >5 cells from at least two independent experiments.
(TIF)

S7 Fig Expression of WT and domain I mutants for RNA filter binding assay. Purified cleaved domain I (35–215) analysed by SDS-PAGE and Coomassie staining (**A**), or Western blot (**B**) with sheep polyclonal antiserum against NS5A.
(TIF)

S8 Fig Summary of the position and potential role of domain I mutants. The two different dimeric conformations of NS5A domain I are shown, “open” (1ZH1) [15] (left, blue/red) and “closed” (3FQM) [16], (right, grey/red). P35 highlighted in aquamarine is located in the P29–P35 interaction loop of NS5A dimers in the open conformation; V67 in green is exposed on the surface of both dimer structures; P145 in burlywood is at the interaction surface of the closed dimer. It is likely that P35 can interact with A92 (orange) from the other monomer that is involved in dimerization of the open conformation. P145 and A146 in the closed dimer face each other across the interaction surface and could possibly exert an effect on dimer interactions.
(TIF)

S9 Fig Lack of NS5A dimerization in intact cells and analysis of effects of mutants on DGAT1 and Rab18 interactions. **A.** A modified version of mSGR-Luc-JFH-1 containing a GFP tag near the C-terminus of domain III of NS5A (termed mSGR-Luc-JFH1(GFP)) was a kind gift from John McLauchlan. In vitro transcribed mSGR-Luc-JFH1(GFP) RNA was electroporated into Huh7.5 cells (lane 1), or Huh7.5 cells stably harbouring the SGR-Neo-JFH1 (lane 2) or SGR-Neo-JFH1(NS5A-OST) [72] (lane 3), or co-electroporated with either pCMV10-NS3-NS5B plasmid (lane 4) or mSGR-Luc-JFH1 RNA (lanes 5, 6) into Huh7.5 cells. Alternatively, DNA constructs of both pCMV10-NS3-NS5B (GFP) (GFP tagged NS5A) and pCMV10-NS3-NS5B were co-transfected into Huh7.5 cells (lane 7). Cells were harvested into GLB at 72 h.p.e. or 48 h.p.t. and subjected to GFP pull down assay following the GFP-Trap® (ChromoTek) protocol. After GFP-Trap, protein bound on beads (lower panel) together with input samples (upper panel) were analysed by Western blot using anti-NS5A antibody. **B.** RNAs were transcribed from mJFH-1 constructs containing the One-Strep tag at the C-terminus of domain III of NS5A (mJFH1-5A-OST) and electroporated into Huh7.5 cells. After purification using the Strep-Tactin system, protein bound resins were subjected to analysis by Western blot using anti-NS5A and anti-DGAT1 (top panel) or anti-Rab18 antibodies (bottom panel).
(TIF)

S10 Fig P35A, V67A and P145A exhibit similar DCV sensitivity to WT NS5A in a genome replication assay. Huh7.5 cells electroporated with the indicated mSGR-Luc-JFH-1 RNAs were treated with serial 10-fold dilutions of daclatasvir (DCV) in duplicate at a final concentration of solvent (DMSO) of 0.25% (v/v), from 4 h.p.e. for 72 h prior to harvest for luciferase assay. Relative luciferase units are expressed as a percentage of DMSO-only treated cells and EC_50_ curves were calculated using Prism 7 (Graphpad).
(TIF)

S1 Table Isolates used for Domain I sequence alignment. Sequences of NS5A amino acids from 29 virus isolates from 7 HCV genotypes and 10 related hepaciviruses were selected from NCBI database for alignment analysis.
(XLSX)

S2 Table Summary of selection of amino acid sites for mutation in NS5A domain I and their phenotypes. 1ZH1 and 3FQM represent two different crystal structures of NS5A domain I. After sequence alignment, all the absolutely conserved residues are listed in the first column. ‘+’ indicates that the residue is on the surface of the domain I or within the zinc-binding motif. ‘++’ means the conserved residues are also the zinc-binding sites. Amino acids that were both surface exposed and out-with the zinc-binding motif were mutated. Cysteine 59, within the zinc-binding motif, was chosen as the positive control as C59A has been documented to be a non-replicative mutant [70].
(XLSX)

S1 Text Supplementary materials and methods. (DOCX)
