# Supplementary figures and images for: A role for domain I of the hepatitis C virus NS5A protein in virus assembly
Source: PLoS Pathog. 2018 Jan 19;14(1):e1006834. doi: 10.1371/journal.ppat.1006834 (PMC5792032; doi:10.1371/journal.ppat.1006834)

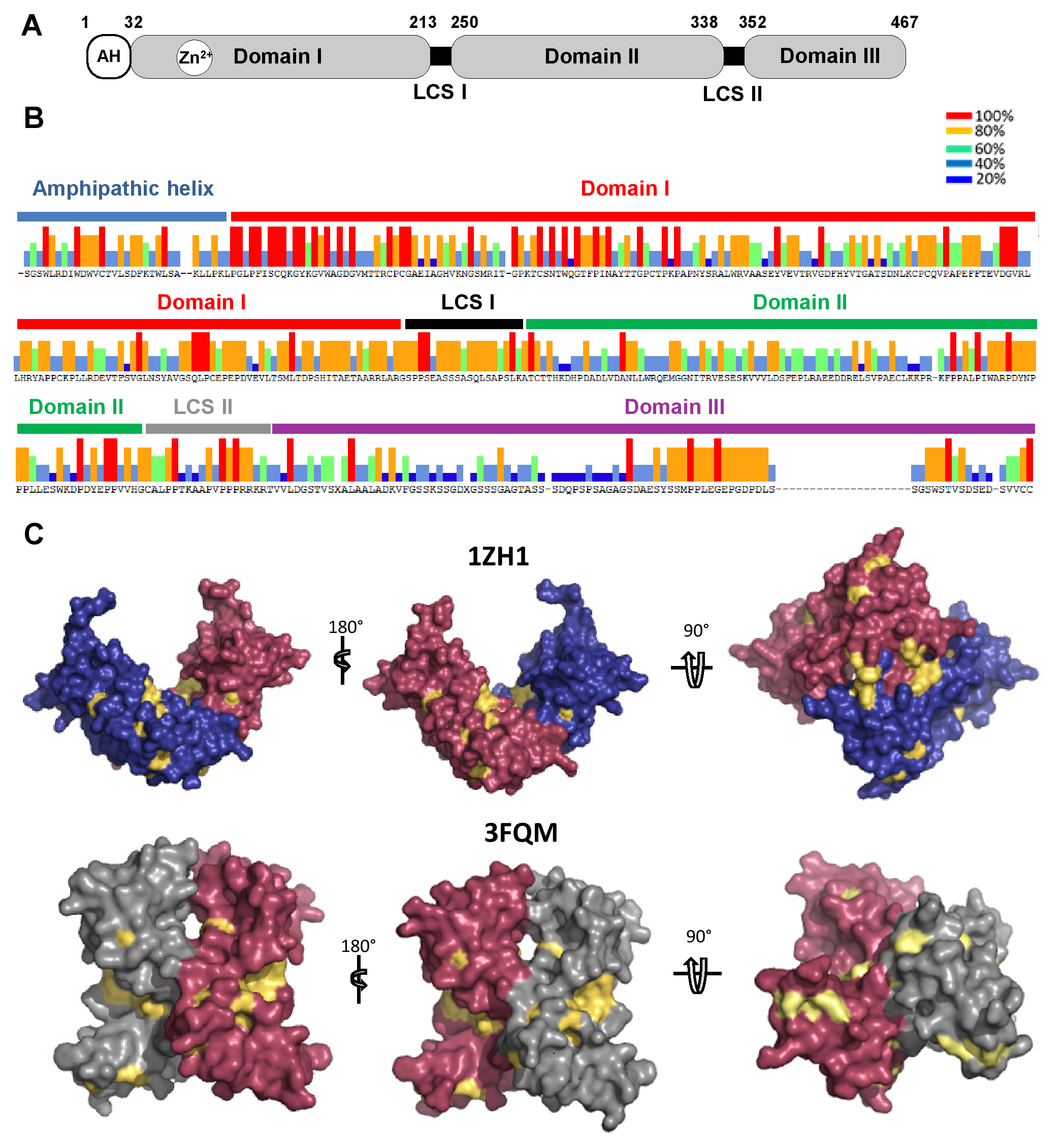

Supplement: S1 Fig — A. Schematic representation of the domain organization of NS5A. The three domains (I-III), the linking low complexity sequences (LCSI and II), and the membrane anchoring amphipathic helix (AH) are illustrated. Numbers indicate positions of amino acids in the JFH-1 genotype 2a NS5A sequence. B. Conservation of three different NS5A domains from HCV isolates representing each genotype and related hepaciviruses. Isolates used for analysis are listed in S2 Table. Filled bars in different colours indicate the percentage conservation at each residue as indicated in the key below. Gaps refer to locations where there are insertions in the JFH-1 sequence, compared to consensus, particularly the 18 amino acid insertion between residues 432–450. C. Analysis of the three dimensional structures of domain I (1ZH1 and 3FQM) using Pymol. Residues highlighted are the conserved amino acids that are located on the surface of two dimeric conformations at positions indicated in S1 Table. (TIF) [file ppat.1006834.s001.tif]

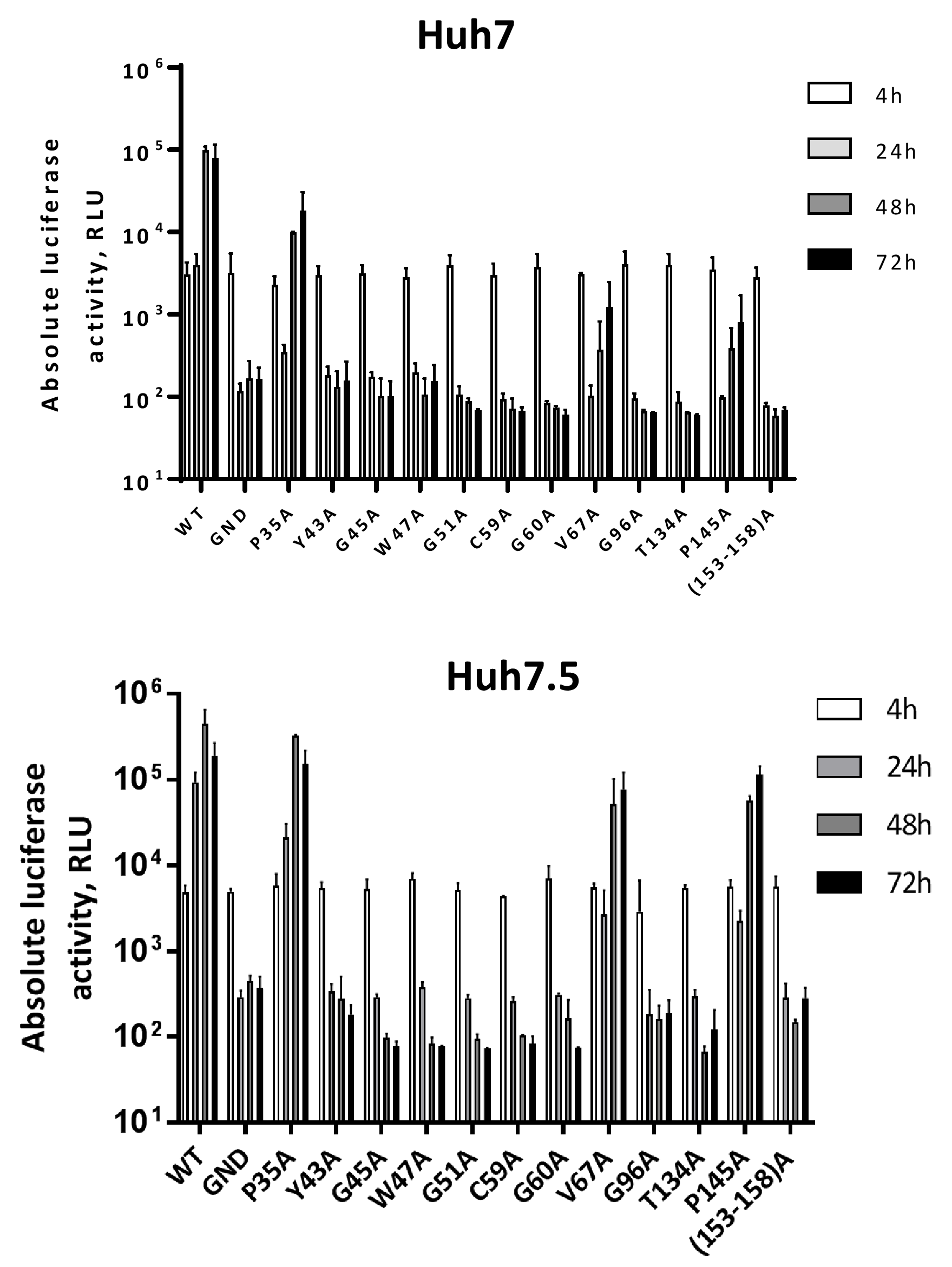

Supplement: S2 Fig — In vitro transcripts of mSGR-luc-JFH-1 containing the indicated mutations were electroporated into either Huh7 (A) or Huh7.5 (B) cells. Luciferase activity was measured at 4, 24, 48 and 72 h post-electroporation (h.p.e.) and plotted as absolute values. 4 h.p.e. values are indicative of input translation and reflect transfection efficiency. Data from three independent experiments are shown and error bars represent the standard error of the mean. (TIF) [file ppat.1006834.s002.tif]

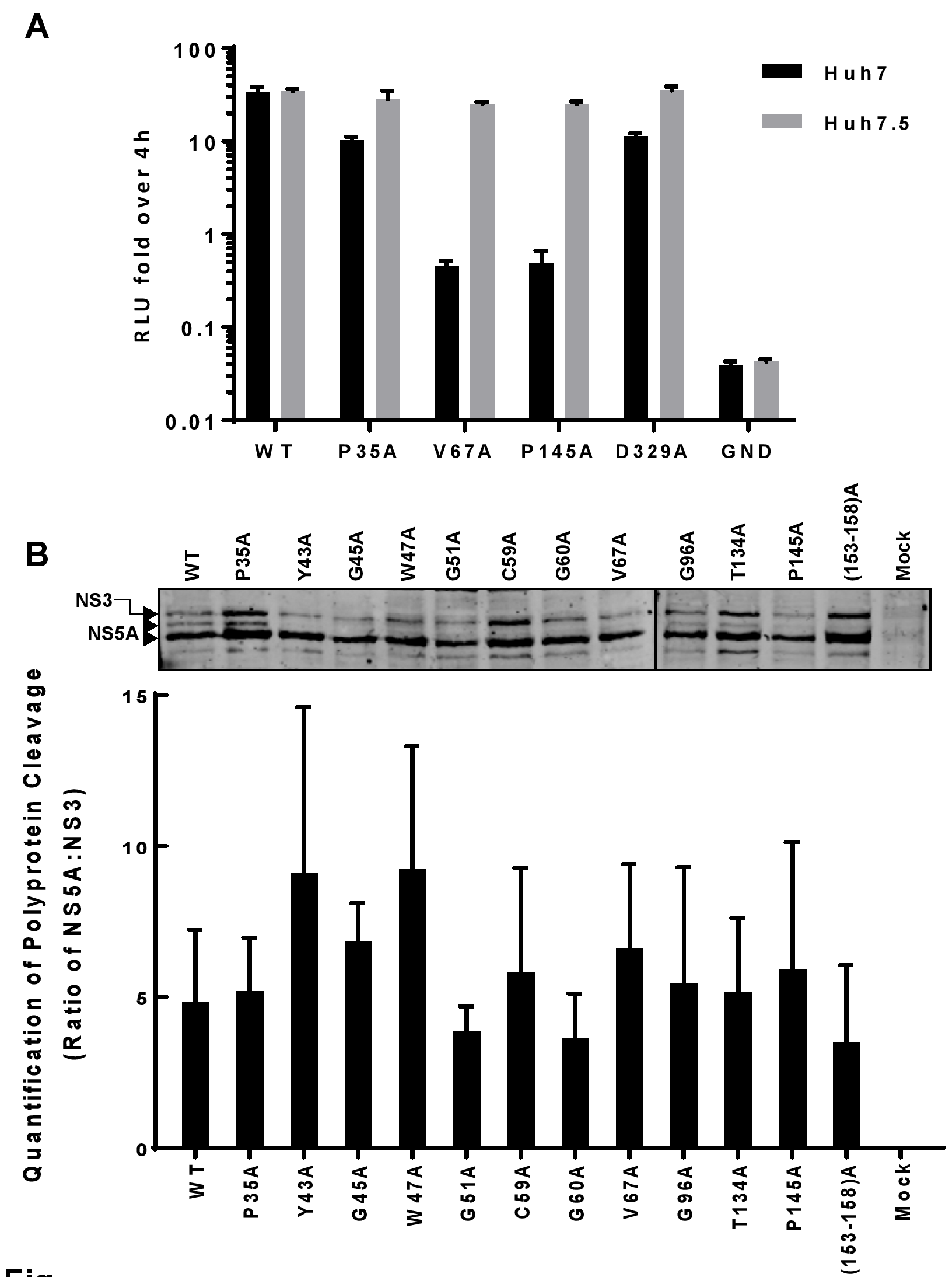

Supplement: S3 Fig — A. WT represents the wild type mSGR-luc-JFH-1. P35A, V67A, and P145A are the mutants of domain I which can replicate at lower levels than WT in Huh7 cells; D329 is located at the C terminus of NS5A domain II. The graph shows the RLU values at 72 h.p.e. expressed as a fold increase over the 4 h.p.e. values. B. Huh7.5 cells were transfected with pCMV10-NS3-NS5B expression vectors containing the corresponding mutations. At 48 h.p.t., cell lysates were harvested in GLB and analysed by SDS-PAGE and Western blotting with anti-NS5A (sheep) and anti-NS3 (mouse). The ratio of NS5A:NS3 was calculated following quantification of Western blot signals using a Li-Cor Odyssey Sa infrared imaging system. Data from three independent experiments are shown and error bars represent the standard error of the mean. (TIF) [file ppat.1006834.s003.tif]

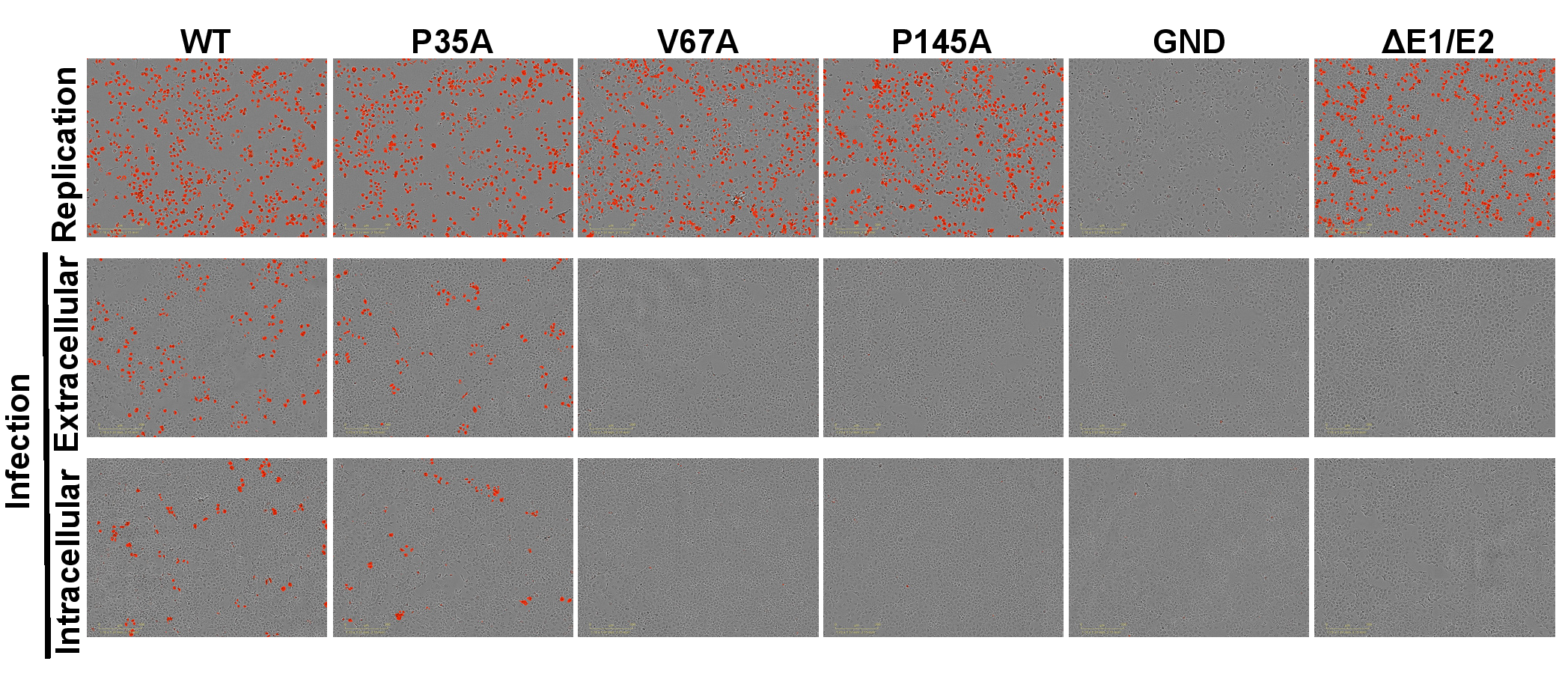

Supplement: S4 Fig — Indirect immunofluorescence analysis for NS5A expression in Huh7.5 cells electroporated with the indicated viral RNAs at 48 h.p.e. (top row). The middle row shows NS5A expression in cells infected with culture supernatants harvested from the cells presented in the top row. Infected cells were analysed at 48 h.p.i. The bottom row shows NS5A expression at 48 h.p.i. in cells infected with cell lysates from the cells in the top row–this represents intracellular virus. After fixation, cells were stained with NS5A antibody and then with Alexa Fluor 568-conjugated donkey anti-sheep IgG (red fluorescence). (TIF) [file ppat.1006834.s004.tif]

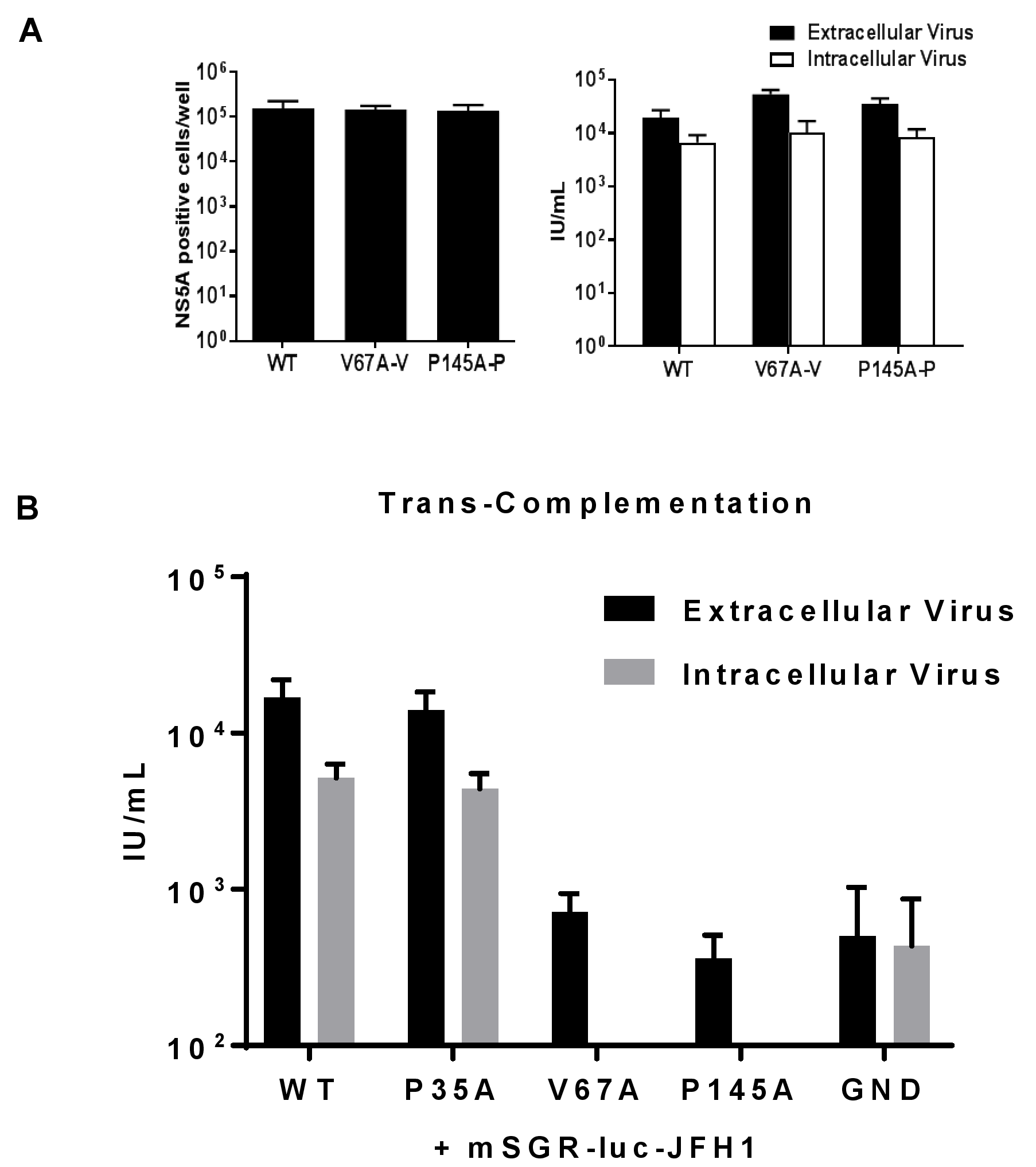

Supplement: S5 Fig — A. Phenotypes of V67A and P145A are not derived from acquisition of an additional compensatory mutation during the cloning process. Revertants were generated by cloning a WT NS5A fragment back into the mJFH-1 V67A or P145A mutant plasmids. Huh7.5 cells were electroporated with in vitro transcripts of the resulting V67 or P145 revertants. Virus genome replication and protein expression was assayed by quantification of NS5A positive cells 48 h.p.e. by using the Incucyte-ZOOM [62]. Intracellular and extracellular infectious virus was titrated at 72 h.p.e. B. In vitro transcribed WT JFH-1 or the indicated mutant RNAs were co-electroporated with the helper RNA (mSGR-Luc-JFH1) into Huh7.5 cells. 72 h.p.e., supernatant was harvested and cells were lysed by repetitive freeze-thaw cycles. Extracellular and intracellular virus was then titrated in Huh7.5 cells and viral infectivity was determined by using Incucyte ZOOM at 48h.p.i. Data from two independent experiments are shown and error bars represent the standard error of the mean. (TIF) [file ppat.1006834.s005.tif]

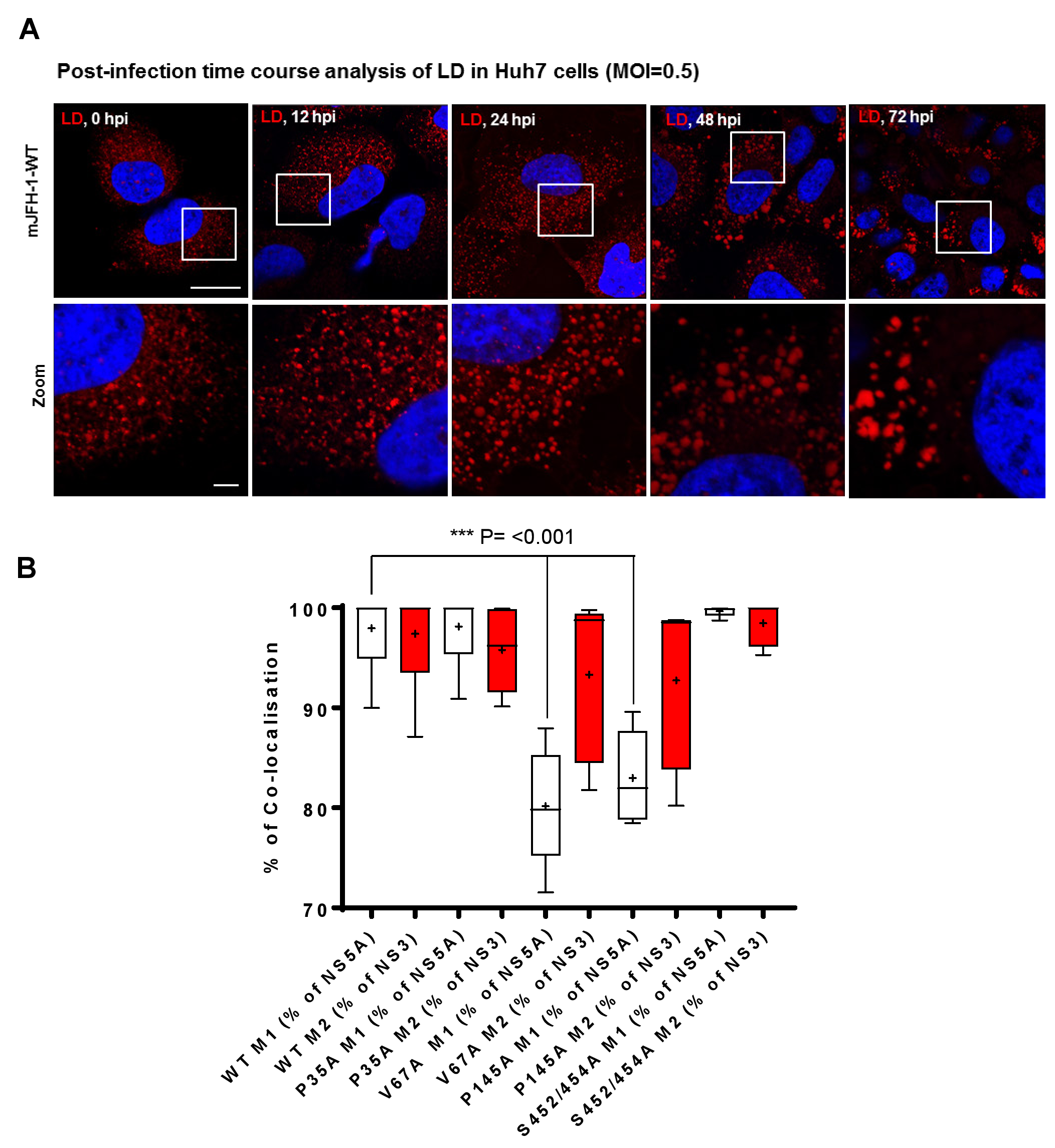

Supplement: S6 Fig — A. Time-course immunofluorescence analysis of LD in HCV infected cells. Huh7 cells were infected with mJFH-1 WT at an M.O.I. of 0.5 ffu/cell. At the indicated h.p.e. cells were fixed and stained with BODIPY 558/568-C12, and DAPI and imaged by Airyscan microscopy. B. Colocalisation of NS5A and NS3. Quantification of the percentages of NS5A colocalized with NS3 (white blocks), or NS3 colocalised with NS5A (red blocks) as shown in Fig 8. Co-localisation calculations were performed on >5 cells from at least two independent experiments. (TIF) [file ppat.1006834.s006.tif]

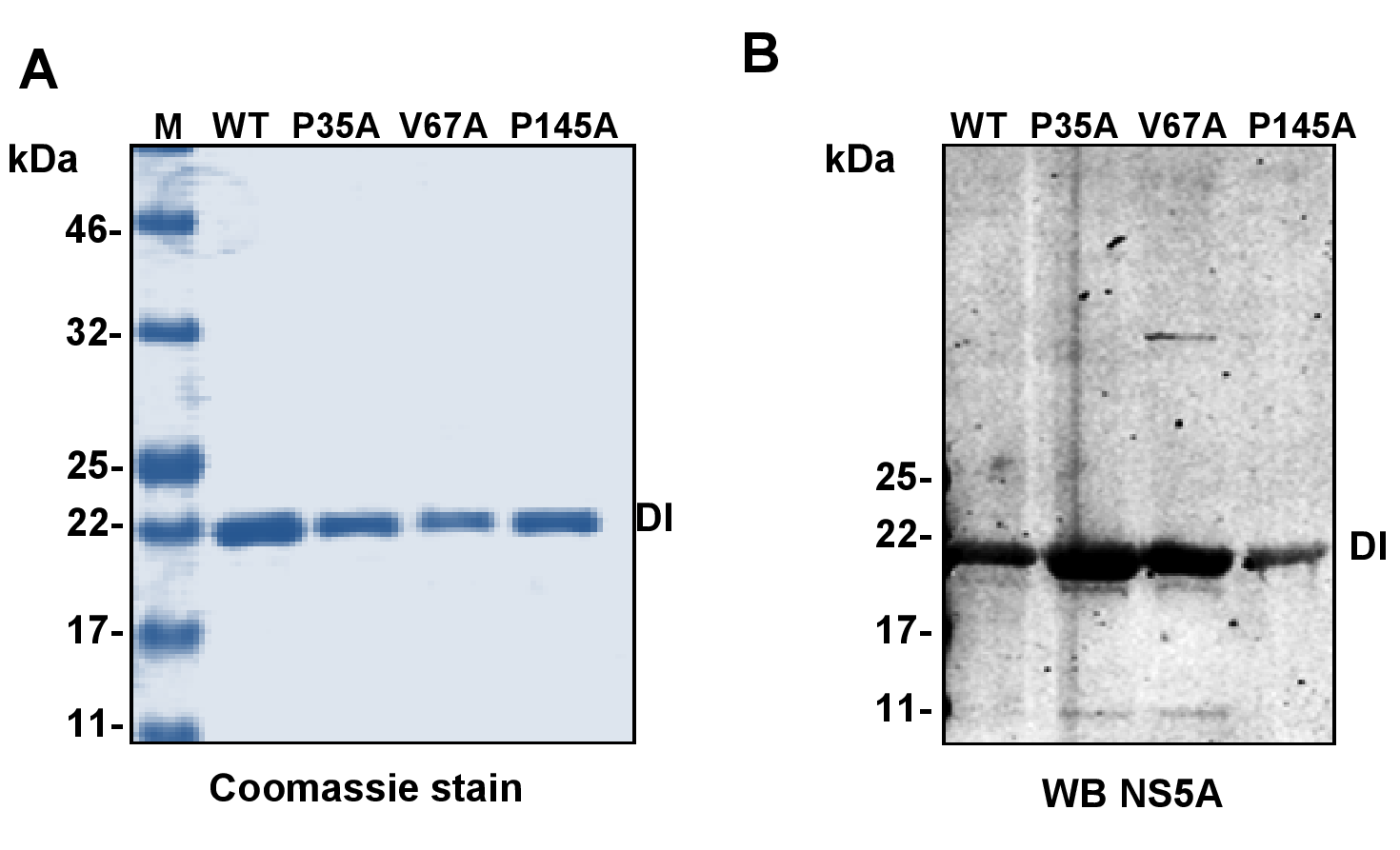

Supplement: S7 Fig — Purified cleaved domain I (35–215) analysed by SDS-PAGE and Coomassie staining (A), or Western blot (B) with sheep polyclonal antiserum against NS5A. (TIF) [file ppat.1006834.s007.tif]

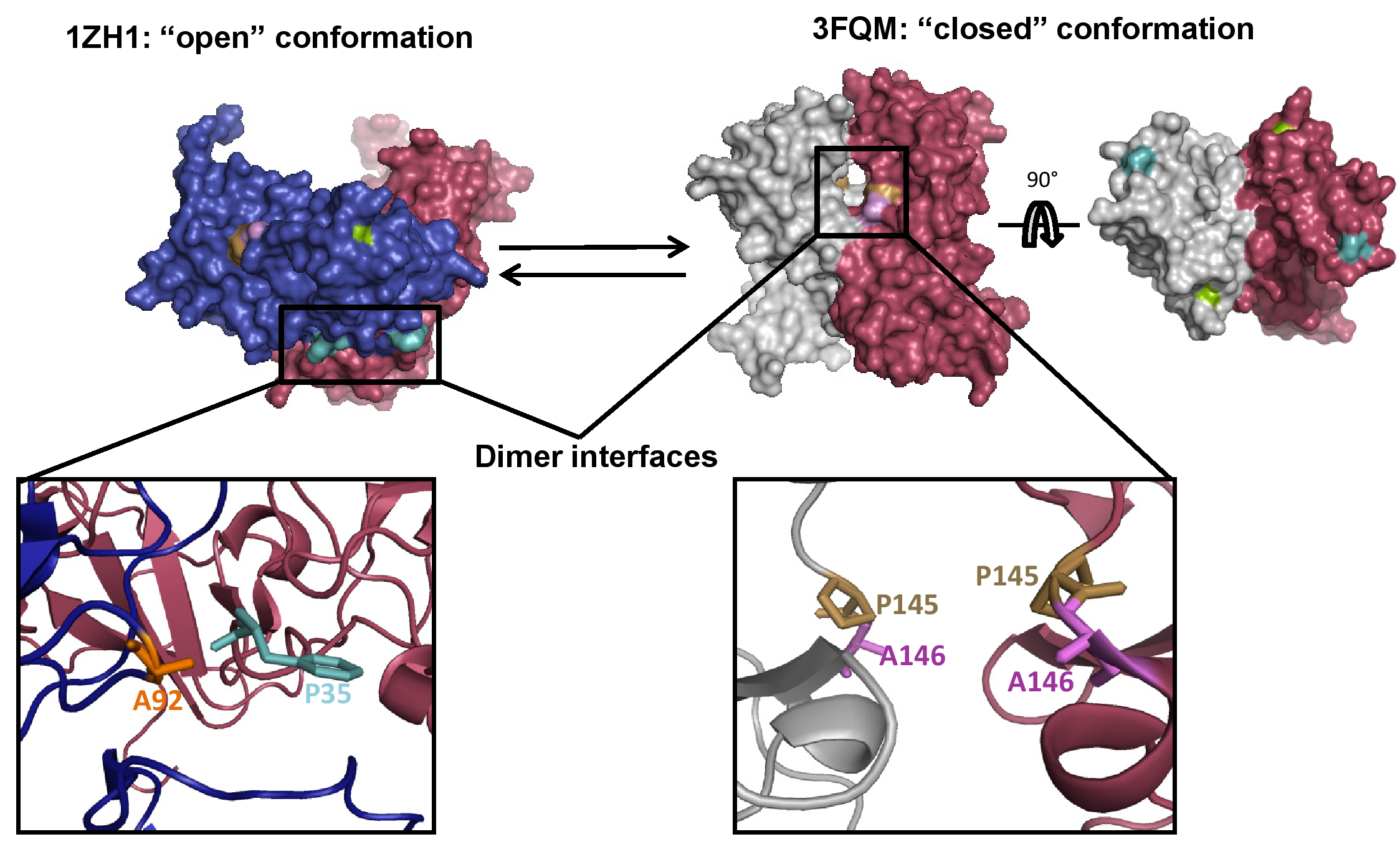

Supplement: S8 Fig — The two different dimeric conformations of NS5A domain I are shown, “open” (1ZH1) [15] (left, blue/red) and “closed” (3FQM) [16], (right, grey/red). P35 highlighted in aquamarine is located in the P29–P35 interaction loop of NS5A dimers in the open conformation; V67 in green is exposed on the surface of both dimer structures; P145 in burlywood is at the interaction surface of the closed dimer. It is likely that P35 can interact with A92 (orange) from the other monomer that is involved in dimerization of the open conformation. P145 and A146 in the closed dimer face each other across the interaction surface and could possibly exert an effect on dimer interactions. (TIF) [file ppat.1006834.s008.tif]

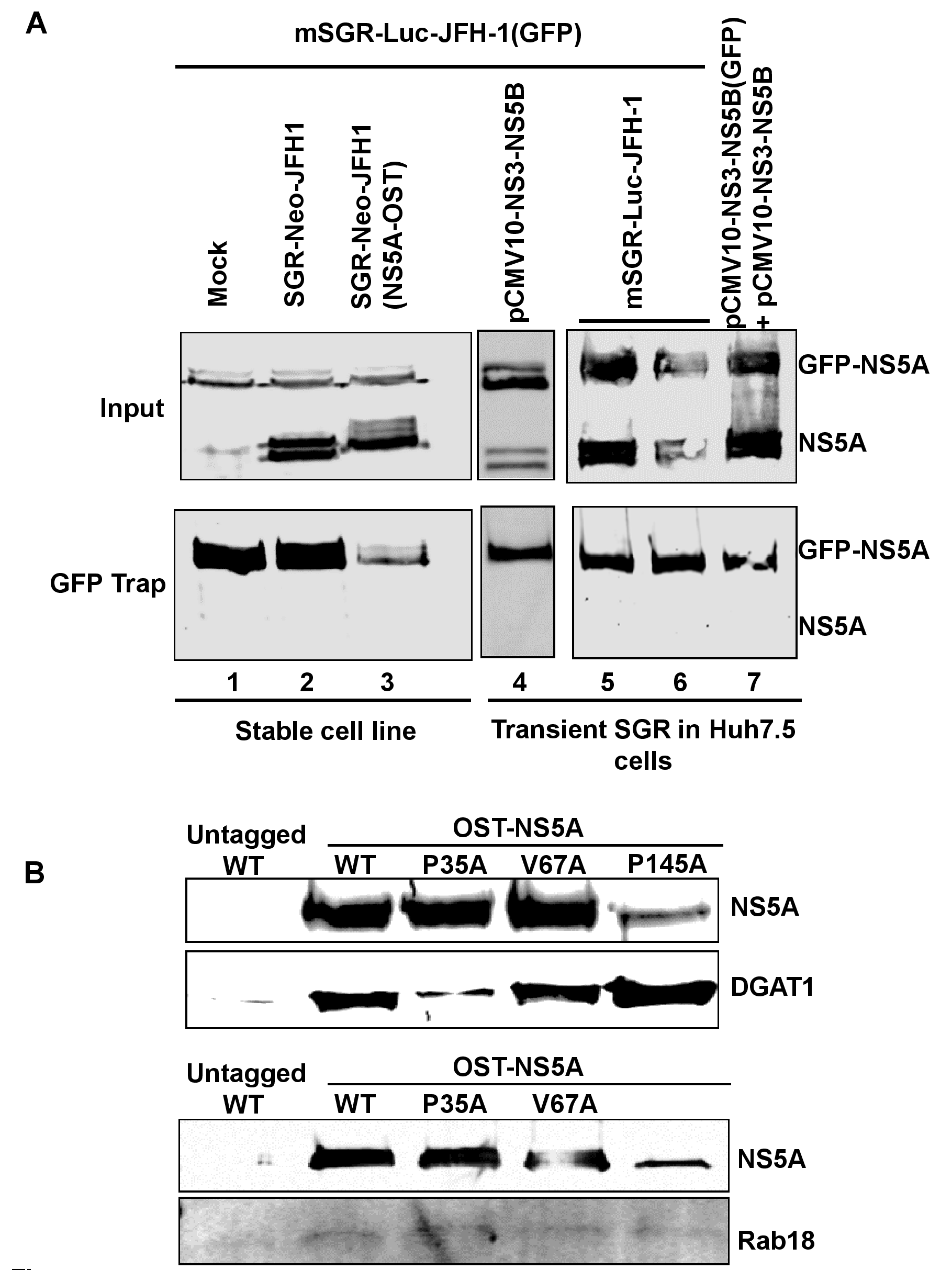

Supplement: S9 Fig — A. A modified version of mSGR-Luc-JFH-1 containing a GFP tag near the C-terminus of domain III of NS5A (termed mSGR-Luc-JFH1(GFP)) was a kind gift from John McLauchlan. In vitro transcribed mSGR-Luc-JFH1(GFP) RNA was electroporated into Huh7.5 cells (lane 1), or Huh7.5 cells stably harbouring the SGR-Neo-JFH1 (lane 2) or SGR-Neo-JFH1(NS5A-OST) [72] (lane 3), or co-electroporated with either pCMV10-NS3-NS5B plasmid (lane 4) or mSGR-Luc-JFH1 RNA (lanes 5, 6) into Huh7.5 cells. Alternatively, DNA constructs of both pCMV10-NS3-NS5B (GFP) (GFP tagged NS5A) and pCMV10-NS3-NS5B were co-transfected into Huh7.5 cells (lane 7). Cells were harvested into GLB at 72 h.p.e. or 48 h.p.t. and subjected to GFP pull down assay following the GFP-Trap® (ChromoTek) protocol. After GFP-Trap, protein bound on beads (lower panel) together with input samples (upper panel) were analysed by Western blot using anti-NS5A antibody. B. RNAs were transcribed from mJFH-1 constructs containing the One-Strep tag at the C-terminus of domain III of NS5A (mJFH1-5A-OST) and electroporated into Huh7.5 cells. After purification using the Strep-Tactin system, protein bound resins were subjected to analysis by Western blot using anti-NS5A and anti-DGAT1 (top panel) or anti-Rab18 antibodies (bottom panel). (TIF) [file ppat.1006834.s009.tif]

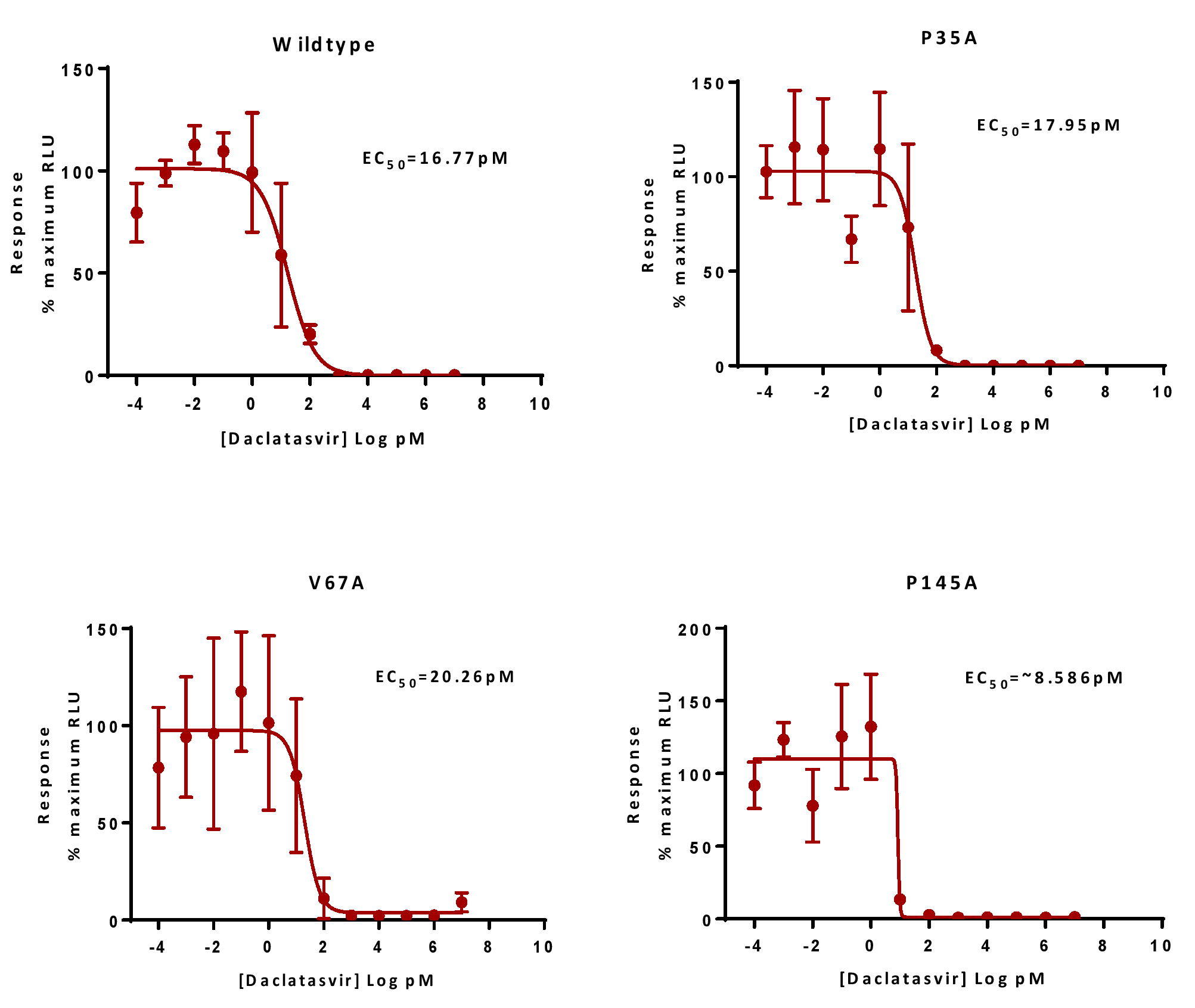

Supplement: S10 Fig — Huh7.5 cells electroporated with the indicated mSGR-Luc-JFH-1 RNAs were treated with serial 10-fold dilutions of daclatasvir (DCV) in duplicate at a final concentration of solvent (DMSO) of 0.25% (v/v), from 4 h.p.e. for 72 h prior to harvest for luciferase assay. Relative luciferase units are expressed as a percentage of DMSO-only treated cells and EC50 curves were calculated using Prism 7 (Graphpad). (TIF) [file ppat.1006834.s010.tif]
