# Supplementary material for: A role for domain I of the hepatitis C virus NS5A protein in virus assembly
Source: PLoS Pathog. 2018 Jan 19;14(1):e1006834. doi: 10.1371/journal.ppat.1006834 (PMC5792032; doi:10.1371/journal.ppat.1006834)
Supplement: S1 Text — (DOCX) [file ppat.1006834.s013.docx]

# A role for domain I of the hepatitis C virus NS5A protein in virus assembly

Chunhong Yin, Niluka Goonawardane, Hazel Stewart^#^, Mark Harris

School of Molecular and Cellular Biology, Faculty of Biological Sciences, and Astbury Centre for Structural Molecular Biology, University of Leeds, Leeds, LS2 9JT, United Kingdom

^#^ Present address: Division of Virology, Department of Pathology, University of Cambridge, Cambridge CB2 1QP, United Kingdom

## Supplementary materials and methods

### Transfection of DNA

pCMV10-NS3-5B constructs were transfected into Huh7.5 cells using FuGene 6 (Promega) transfection reagent according to the manufacturer’s directions. For example, Huh7.5 cells were seeded at 2 x10^5^ cells/well in a 6-well dish 24 h prior to transfection. Transfection reaction was prepared by adding 1 µg plasmid DNA to 98 µl Opti-MEM (Life technologies) followed by the addition of 3 µl FuGene 6, gentle mixing and incubation at room temperature (RT) for 30 min. Transfection reaction was then added drop-wise to cells in 6-well plates. Opti-MEM was changed for DMEM/10% foetal calf serum at 6 h.p.t. Typically at 48 h.p.t., cells were washed twice in PBS and lysed in 50 µL Glasgow lysis buffer (GLB) [1% Triton X-100, 120 mM KCl, 30 mM NaCl, 5 mM MgCl_2_, 10% glycerol, and 10 mM piperazine-N,N’–bis (2-ethanesulfonic acid) (PIPES)-NaOH, pH 7.2] supplemented with protease inhibitors and phosphatase inhibitors (Roche Diagnostics).

### Constructions and purification of NS5A domain I for GST-pulldown assay

DNA fragments flanked with *Bam*HI and *Xho*l restriction sites encompassing amino acids 35 to 249 of NS5A were amplified by PCR using wildtype or mutant JFH-1 SGRs as templates (primer sequences available upon request). PCR products were cleaved with *BamH*I and *Xho*l and cloned into either pGEX6P-2 or pET28a-SUMO vectors to allow expression of domain I N-terminally fused to either GST or a His-SUMO affinity tag. The resulting plasmids were transformed into cultures of *Escherichia coli* BL21 (DE3) pLysS, which were grown to OD_600_ 0.6-0.8 at 37°C and induced with 100 μM isopropyl β-D-1-thiogalactopyranoside (IPTG) at 18°C for 6 h. Cells were recovered by centrifugation and resuspended in 20 mL GST-DI lysis buffer as described previously [[7](#_ENREF_7)] and His-SUMO-Domain I (His-SUMO-DI) lysis buffer [binding buffer (100 mM Tris pH 8.2, 200 mM NaCl, 20 mM imidazole) supplemented with 40 μl DNase, 40 μl RNaseA, 2 mg/ml Lysozyme and protease inhibitors (Roche)] per 1 L of pelleted culture. Cells suspensions were placed on ice for 30 min and lysed by sonication on ice at amplitude of 10 microns for 12 pulses of 20 sec separated by 20 sec. The extract was clarified by centrifugation at 18,000 rpm for 30 min at 4°C twice and then the supernatant was filtered through a 0.45 µm filter. GST-DI protein was applied to glutathione Sepharose 4B resin (GE Healthcare) following the manufacturer’s instructions. His-SUMO-DI protein samples were applied to wash buffer equilibrated HisTrap column (GE Healthcare). The column was washed 3 times with 5 column volumes binding buffer and eluted using the same buffer with 250 mM imidazole.

### Constructions and purification of NS5A Domain I for RNA filter binding assay

Wild type and mutated domain I DNAs spanning from residues 35 to 215 of NS5A were amplified (primer sequences available upon request) and cloned into pET-28a-SUMO vector with a SUMO protease cleavage site (-GG-) at the N-terminal end of domain I to produce the native protein. Purification of His-SUMO-Domain I proteins followed the protocol described above but were dialyzed into dialysis buffer [20 mM Tris-HCl, pH 8.2, 150 mM NaCl and 10% (v/v) glycerol] prior to addition of SUMO protease. After SUMO protease cleavage, the His-SUMO tag and SUMO protease were removed from the cleavage sample by passing it through a second HisTrap column. The flow-through and wash fractions were pooled and concentrated using 10 kDa molecular mass centrifugal concentrators (Merck) and stored at -80 °C after flash freezing in liquid nitrogen.

### Purification of NS5A-OST

In vitro transcribed mJFH-1-5A-OST RNAs were electroporated into Huh7.5 cells and seeded in 10 cm x10 cm dishes. At 72 h.p.e., cells were washed twice in PBS, scraped and pelleted at 1500 g for 5 mins at RT, before lysis in GLB (1 ml per dish). Lysates were clarified by centrifugation at 10,000 g for 5 min at 4°C. Supernatants were applied to 100 µL of Strep-Tactin Sepharose for batch affinity purification of NS5A-OST following the manufacturer’s instructions. After incubation overnight at 4°C, the mixtures were washed three times with wash buffer (100 mM Tris-HCl, pH 8.0, 150 mM NaCl 1 mM EDTA). Bound protein was eluted with 50 µL of 1xSDS-PAGE loading buffer and heated for 10 min at 95°C for Western blot analysis.

### Treatment with daclatasvir (DCV)

Huh7.5 cells were washed twice in cold phosphate-buffered saline (PBS) before electroporating 4x10^6^ cells in cold PBS with 2 µg of mSGR-luc-JFH1 RNA at 975 µF and 260 V. Cells were resuspended in complete media before being seeded into 96-well plates at 3x10^4^ cells/well,. Daclatasvir (DCV) was diluted from a stock of 40 mM in DMSO and applied to cells from 10 µM-0.1 fM at 4 h.p.e. At 72 h.p.e., medium were removed and washed 3 times in PBS followed by addition of 30 µl PLB. Luciferase activity was determined using BMG plate reader by adding 50 µl LAR-1 reagent and recording light emission over 6 seconds. Data were modelled using the model of log (agonist) vs. response model and EC_50_ were calculated through Graphpad Prism 7.
